# Avoiding transcription factor competition at promoter level increases the chances of obtaining oscillation

**DOI:** 10.1186/1752-0509-4-66

**Published:** 2010-05-17

**Authors:** Andreea Munteanu, Marco Constante, Mark Isalan, Ricard V Solé

**Affiliations:** 1 ICREA-Complex Systems Lab, Universitat Pompeu Fabra (PRBB-GRIB), Dr Aiguader 88, 08003 Barcelona, Spain; 2 EMBL-CRG Systems Biology Research Unit, Centre for Genomic Regulation (CRG), UPF, Dr Aiguader 88, 08003 Barcelona, Spain; 3 Santa Fe Institute, 1399 Hyde Park Road, Santa Fe NM 87501, USA

## Abstract

**Background:**

The ultimate goal of synthetic biology is the conception and construction of genetic circuits that are reliable with respect to their designed function (e.g. oscillators, switches). This task remains still to be attained due to the inherent synergy of the biological building blocks and to an insufficient feedback between experiments and mathematical models. Nevertheless, the progress in these directions has been substantial.

**Results:**

It has been emphasized in the literature that the architecture of a genetic oscillator must include positive (activating) and negative (inhibiting) genetic interactions in order to yield robust oscillations. Our results point out that the oscillatory capacity is not only affected by the interaction polarity but by how it is implemented at promoter level. For a chosen oscillator architecture, we show by means of numerical simulations that the existence or lack of competition between activator and inhibitor at promoter level affects the probability of producing oscillations and also leaves characteristic fingerprints on the associated period/amplitude features.

**Conclusions:**

In comparison with non-competitive binding at promoters, competition drastically reduces the region of the parameters space characterized by oscillatory solutions. Moreover, while competition leads to pulse-like oscillations with long-tail distribution in period and amplitude for various parameters or noisy conditions, the non-competitive scenario shows a characteristic frequency and confined amplitude values. Our study also situates the competition mechanism in the context of existing genetic oscillators, with emphasis on the Atkinson oscillator.

## Background

In the relatively young field of synthetic biology [1,2], there is increasing interest in the conception and construction of genetic circuits that are reliable with respect to their designed function. Having given the first step with the implementation of biological switches [3], the next step for synthetic biology was the construction of biological oscillators. The first successful implementation [4] constituted the onset of the quest for oscillators of tunable amplitude and/or period. Oscillators are important in biology for many reasons, since they are involved in the cell cycle, cell motion, embryonic development [5]. In some cases, the genetic machinery associated with the oscillatory behavior is rather small.

In the context of genetic designs, two- and three-element networks have been shown to be enough for implementing oscillatory behavior either of transient or sustained type [4,6]. Several theoretical and experimental implementations of suggested robust tunable oscillators exist [7,8]. However, predictable robustness of genetic networks is still a difficult task. The unpredictability of synthetic designs originates from the more-than-additive effect of assembling multiple building blocks, and has raised philosophical conundrums [9]. Intuition alone cannot grasp the effects of multiple regulatory interactions and thus mathematical models are particularly well suited for unraveling the implications of the underlying nonlinear interactions [10-14].

The way towards understanding and reliably predicting the information encoded by combinations of cis-regulatory sites lies in the coupling of such mathematical models with the experimental synthesis of regulatory promoter libraries [15-17]. Recent studies address the impact of some particular designs on the expected patterns of genetic switches and clocks [18,19]. The authors of these studies have shown that the interplay between rate constants and circuit structure leads to nontrivial outcomes. Moreover, these studies suggest different views of how to explain the observed behavior. But several aspects have not yet been properly addressed, in particular the importance of the architecture of promoters and the resulting behavior.

In this paper, we show how the dynamics of a simple two-component activator-inhibitor oscillator is drastically affected by the architecture of the promoters and the nature of binding by the transcription factors. We show that competitive DNA-binding of several transcription factors acting at the level of a promoter leads to a more fragile oscillator than with non-competitive binding. The type of binding mechanism and the circuit architecture together determine the features of the resultant oscillations. The intention of the present work is to emphasize the importance of choosing the appropriate mathematical modeling in ensuring the experimental achievement of the desired biological function.

We also argue that detailed studies of these DNA-binding mechanisms could provide new insights into old experimental and theoretical studies of genetic circuits. A clear example is the experimental implementation of the Atkinson oscillator [6] for which several distinct mathematical models (and thus distinct dynamical behaviors) exist in the literature. Which among these models would be most suitable with respect to Atkinson's experimental implementation still remains to be determined, as the details of the biological binding mechanism are not sufficiently understood.

This paper is organized as follows: *Background *section continues with an extended introductory section on transcriptional regulation and genetic circuits that provides the framework of discussion of the existing models and experimental implementations. *Results and discussion *section details the main results on the competitive and non-competitive transcriptional binding, especially the mathematical characteristics of the route towards sustained oscillations. In *Conclusions *section we emphasize the implications of the DNA-binding mechanism on the reliability of the genetic circuit in terms of its desired function.

### Genetic circuits

Genetic circuits involve a certain number of genes that regulate one another's expression [20]. This regulation is achieved by means of the RNA or protein they encode for, that serve as regulators for other genes' expression. As major regulators, the protein factors influence the rate of transcription, translation or post-translational modification. RNAs, too, can have a variety of regulatory functions (i.e translation inhibition by microRNAs). The regulatory mechanisms are still in the process of being elucidated [21] and have been already addressed in synthetic biology [22,23].

Among the reasons for constructing synthetic biological circuits, there is the goal of understanding the fundamental building blocks and regulation mechanisms of biology [24,25], and subsequently manipulating and monitoring biological processes at the DNA level [26,27]. The majority of the existing works in systems and synthetic biology have focused on transcriptional regulation, as the interactions are relatively modular and can be altered at will. Additional File 1 constitutes an appendix on the basics of this regulation mechanism and the definition of the Hill function, which describes transcriptional interactions. Comparatively few studies have focused on other types of regulation, such as the post-transcriptional regulation employed in Fussenegger's Lab [8,28]. A combination of transcriptional regulation and post-translational modification is common in the literature, where the latter refers to a nonlinear degradation of a transcription factor [18,29]. In general, this nonlinear degradation involves a saturated response [30-32], sometimes referred to as enzymatic control. The term of enzymatic control was inherited from the first studies on chemical circuits [33-36] which could be considered as the *in silico *progenitors of today's genetic circuits. Moreover, the coupling of transcriptional regulation with metabolic flux [37] has opened yet another door to innovation and control of biological circuits. Even though much work is needed in terms of reliability and control of any individual oscillator design, there are already advances towards the next step: coupling of genetic oscillators [38,39]. This step allows accessibility to new behaviors that are not possible at the single-oscillator level, similar to the introduction of spatial features into homogeneous systems [40]. Given all these distinct mechanisms and types of control, one should intuitively expect that not only the "*sign*" of genetic interaction (positive or negative), but also the *type *of interaction (transcriptional, post-transcriptional, post-translational) will affect the behavior of the circuit. These dynamic consequences have only begun to be explored and are far from being clarified.

When referring to an interaction network, one considers two types of interactions or loosely called feedbacks: positive (protein A promotes the production of protein B) and negative (A inhibits the production of B). Beyond the details of the interaction itself, the theory of circuits associates an overall sign to the interaction network itself, with the circuit being positive or negative depending only on the parity of the number of negative interactions in the network. A network is positive if it contains either positive interactions alone, or an even number of negative ones, while a negative network has an odd number of negative feedbacks [49,50]. The general theory of circuits has demonstrated that a positive network is a necessary (though not sufficient) condition for multistability (and thus differentiation), while a negative one, for homeostasis (see Ref. [10] for a review). More precisely, it was proved [51] that there is a qualitative difference in the behavior of circuits containing even or odd negative feedbacks, respectively, with the latter showing the possibility of sustained oscillations. A negative feedback alone produces a stable state, unless there exists a sufficiently long time delay, in which case oscillations are possible. In the case of a genetic circuit with a positive feedback, sustained oscillations are not possible even with time delay (not proved though, but reasonably argued [52]).

**Figure 1:**
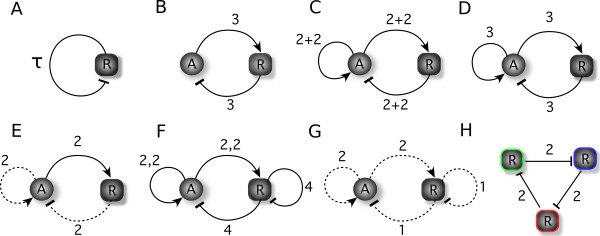
**Transcriptional genetic circuits from the literature**. Figure illustrating the circuit architectures associated to Table 1. In the graphical representation, we have made the distinction between non-competition and competition models by solid and dashed arrowed lines, respectively. In the notation *n*_1_, *n*_2_, ... characterizing the interactions in the schematic figures, the comma separates various binding sites and the degree of the multimer binding to that site. The notation *n*_1 _+ *n*_2 _is employed only for subfigure (c) - see discussion in the Table 1.

The situation might be different in non-genetic feedback loops, for example enzymatic circuits. This is the case commented by Smolen et al. [53] regarding the enzyme phosphofructokinase that shows oscillations in a non-delay (non-genetic) positive loop with limiting rate-supply of precursors. As mentioned above, we are mainly interested in studying the transcriptional interaction, thus we shall not discuss the known circuits that combine transcriptional control with enzymatic one [29,30,32,54], nor shall we comment for the moment on the mRNA-level (post-transcriptional) regulation circuits [8]. Thus, the models discussed in the current study employ only transcriptional regulation. The circuits studied by previous works are schematically represented in Figure 1 and discussed in Table 1.

**Table 1:** Table associated to Figure 1 representing known genetic oscillators that employ transcriptional regulation alone.

Index	Bifurcations analysis	Reference of the study and remarks	Competition
**(A)**	Hopf bifurcation	*Analytic: *Lewis (2003) [41] citing Glass&Mackey (1988) [5] *Experimental: *Swinburne et al. (2008) [42]	-

**(B)**	Hopf bifurcation	*Analytic: *Widder et al. (2007) [43]. The minimum Hill exponent to make oscillations possible is *n *= 3.	-

**(C)**	-	*Experimental: *Atkinson et al. (2003) [6]: The notation 2 + 2 refers to the fact that the DNA loops operate when 2 DNA-bound dimers form a tetramer. It is still not clear if the design functions with competition or not, and this is the reason for employing the 2 + 2 notation instead of the arrows.	Probable

**(D)**	-	*Numeric: *Scott et al. (2006) [44] on the Atkinson oscillator.	No

**(E)**	SNIC bifurcation	*Numeric: *Guantes&Poyatos (2006) [18] associate their Design I to the Atkinson experimental context. The oscillators they propose due their oscillations to the time-scale difference between activator and repressor life-time. Otherwise, higher multimers than dimers are needed.	Yes

**(F)**	Hopf bifurcation	*Numeric: *Hasty et al. (2002) [45] *Experimental: *Stricker et a. (2008) [7]	No

**(G)**	Hopf bifurcation	*Numeric: *Smolen et al. (1998) [46]. The activator needs to be at least a dimer for the existence of the oscillations.	Yes

**(H)**	-	*Experimental and numeric: *Elowitz&Liebler (2000) [4]: *n *= 2 from Figure (1) above makes reference to the value employed by Elowitz&Leibler in their model. See also the discussion in the Supporting Information from Buchler et al. (2005) [47].	
	Hopf bifurcation	*Analytic: *Mueller et al. (2006) [48]: a general case.	-

#### Oscillations from negative feedbacks alone

The simplest genetic circuit capable of producing sustained oscillations is a circuit of one gene with delayed self-inhibiting regulation (Figure 1A). The theoretical analysis of such systems has been performed long before the experimental advances contemplated the measurements and implications of transcriptional and transport delays [55]. More recently, stochastic models [41,56] have added to these deterministic ones, showing that noise promotes the oscillatory behavior in a delayed negative circuit. Due to its simplicity, the case of self-inhibitory feedback with delay has been addressed in synthetic biology as well [42]. Beyond synthetic circuits, transcriptional and transport delay are expected to be more relevant in eukaryotes than in prokaryotes, and thus can and have been assigned as the causal factor of oscillatory behavior in only a few specific mammalian genetic networks, all composed of short negative feedback loops [57]. Nevertheless, the relation between delay, protein half-life and cooperativity required for oscillatory solutions (see page 192 of Ref. [5] for the analytical relation) is not easily fulfilled under normal conditions, and this is also the reason for not considering these delays in the general-purpose modeling of genetic circuits.

As discussed above, a circuit of odd-number negative feedbacks is equivalent to a positive-and-negative feedback circuit, and thus oscillations are theoretically possible. For example, a three-gene synthetic oscillator based on (non-explicitly delayed) negative interactions alone has been already verified experimentally (the "repressilator" [4]; Figure 1H) and its generalization has been studied theoretically [48]. The clarification of the specific parameter ranges for which oscillations occur is crucial for the design of a synthetic circuit, such as the repressilator [4]. However, it is an issue rarely considered. And the subsequent experimental challenge is to achieve these parameter ranges in the experimental circuit. For this purpose, the repressilator implementation required extreme solutions [47]: very strong promoters, very high degradation rates of the proteins and multiple binding sites to increase the nonlinearity of the interactions (that is, increase the Hill coefficient - see Additional File 1).

## Results and Discussion

### On two-component genetic oscillators

To achieve the goal of reliable synthetic circuits, several strategies have been employed by the community. One strategy is the *in silico *evolution of biochemical and genetic networks based on simplified principles of biological interactions and where the fitness function rewards circuits characterized by the function required to be implemented [58,59]. Another strategy is to bring together experimental data and modeling framework in search of designs that are tunable and robust to fluctuations [16,60,61]. Yet another strategy of a more systems-biology flavor points towards understanding the dynamics of minimal fundamental modules [18,32,43], and the current work belongs to this approach.

Sufficient conditions for the occurrence of sustained oscillations are still an open issue in nonlinear dynamics theory, and naturally they are continuing to be delineated in feedback-based gene networks [10]. It is clear though that the combination of both negative and positive feedback is a necessary (but not sufficient) condition in order to obtain oscillations for (non-explicitly delayed) two-gene circuits [43]. Analytically, the simplest genetic oscillator of two genes needs two feedbacks: a positive and a negative one (see Figure 1B). However, this might not be the easiest system from the experimental point of view, due to the high value of cooperativity (Hill exponent *n *≥ 3) required for sustained oscillations (i.e. non-damped). For a multimer binding to a single binding site, the Hill coefficient coincides with the multimer's degree (i.e. *n *= 2 if the TF is a dimer; *n *= 4, if it is a tetramer). From the experimental point of view, one hopes to obtain a high Hill exponent by introducing several binding sites for the transcription factor (TF). In this way and depending on the binding affinities of the TF, binding cooperativity *may *occur, leading to a higher exponent than the one corresponding to the degree of the multimer employed. For example, a Hill coefficient of *n *= 3 as in the example above may be obtained with dimers binding to two sites if some degree of cooperativity exists [62,63].

**Figure 2:**
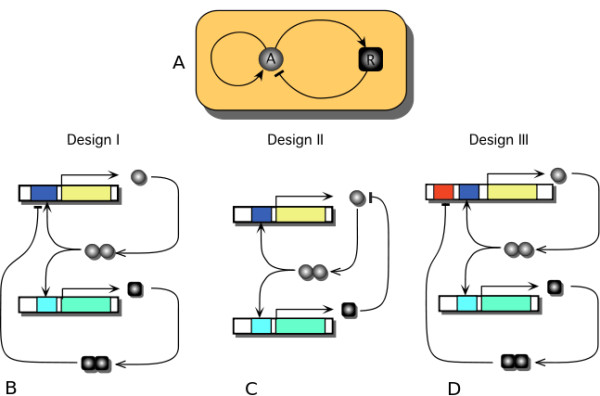
**Distinguishing competition versus non-competition of transcription factors at promoter level in a chosen circuit architecture**. Three different implementations compatible with the scheme in (A): Design I - inhibition through competition at the activator promoter (B), Design II -inhibition through active degradation of the activator by the repressor (C), and Design III - specific inhibition *(d)*. The cases (B) and (C) are discussed in [18], while there is controversy whether the Atkinson oscillator [6] is based on case (B) [18] or (D) [44].

Figure 1C, D, E refer to a genetic *relaxation-based *oscillator containing three interactions: two positive and one negative. When the expression of a TF, say protein **A**, is controlled by two TFs, say **A **and **R**, through a positive and a negative feedback, respectively, one can imagine several transcriptional scenarios. In Figure 2 we illustrate several implementations compatible with the schematic representation of the circuit shown in (*A*). For the competition case shown in (*B*), the activator **A **(circle) and the repressor **R **(square) compete for the binding on the promoter in the activator's module. In this case, the transcription of the activator occurs from two possible states of the activator promoter: occupied with **A **or occupied with **R**. For the design in (*C*), the inhibition occurs through post-translational control: the degradation of the activator is catalyzed by the repressor. These two designs have been studied by Guantes&Poyatos (2006) [18] and we follow here their terminology: Design I and Design II, respectively.

We introduce in Figure 2D Design III which confers to both TFs their binding sites. This implies that the transcription of **A **results from several combinations of the A promoter: *AX*, *XB*, *AB*, with *X *implying free site. Compared to Design I, the mathematical form of Design III is closer to the model associated to the Atkinson oscillator introduced by Scott et al. [44]. Guantes&Poyatos [18] claim that Design I corresponds to Atkinson model. And indeed Atkinson et al. [6] envision that, even though the experimental implementation is similar to Design III from Figure 2, the resultant activation DNA loop and the repression DNA loop would be mutually exclusive [64]. This exclusion might thus imply a competition process, leaning towards effective similarity to Design I. From the experimental perspective, the data has revealed *damped *oscillations rather than the sustained ones expected from the model [6]. There is still no clear identification of the cause of damped oscillations, and no definite study on the extent of the exclusion between the two DNA loops.

Below we shall assess the implications of binding competition by comparing Design I with Design III. Before detailing these results, we wish to establish a framework for their comparison in the light of two existing works: Guantes&Poyatos [18] and Conrad et al. [19]. Guantes&Poyatos [18] argue that there is a fundamental difference, in terms of dynamic behavior, between Design I and Design II. The number of steady states in the two designs and their stability properties completely determine the onset of *sustained *oscillations. The way these equilibria lose their stability, that is the underlying bifurcation type, determines the type of oscillators. More precisely, when a parameter of the system is varied, a *bifurcation *is said to occur at a certain parameter value (bifurcation point) for which a qualitative change in the behavior of the system appears, such as the birth of sustained oscillations [65,66].

We suggest that circuit architecture AND DNA-binding processes determine the type of bifurcations leading to oscillatory solutions, types that confer specific features to the oscillations. Previous results show that Design I induces oscillations with large activator amplitudes and arbitrarily small frequencies, and acts as an "integrator" of external stimuli, while Design II shows emergence of oscillations with finite, and less variable, frequencies and smaller amplitudes, and detects better frequency-encoded signals ("resonator"). This classification originates from neuronal oscillations where the type of neuron (type I or type II) determines entrainment properties, phase response curves and robustness to noise [67]. In a nutshell, Guantes&Poyatos [18] suggest that the underlying biochemical mechanism of repression may determine whether the genetic circuit behaves as a resonator or as an integrator. This statement is essentially correct, as the mathematical difference between the two models makes the dynamical behavior possible. On the other hand, Conrad et al. [19] argue that the rate constants rather than the biochemical mechanism have the capacity to determine which type of behavior is observed. They show that for a certain parameter interval, Design II passes initially through an integrator-originating bifurcation and subsequently (higher parameter values) through a resonator-type bifurcation. Even though the parameters are responsible for the occurrence of these bifurcations, it is nevertheless the underlying mechanism (how the architecture is biologically implemented) that makes them possible. In our comparative study, we show that indeed by changing different parameters, we allow different types of oscillations-originating routes, but we also show that the biological regulation mechanisms make them possible.

#### To compete or not to compete?

In terms of equations, the systems describing the implementations from Figure 2, following the notations from Guantes&Poyatos [18], are:

With  ≡ *dx*/*dτ*, and the rest of the variables and parameters being defined in Table 2. As the details on how Design I and II were obtained and the parameters' definition are extensively presented in the Supplementary File of [18], we have chosen to include in the Additional File 1 only the necessary details for recovering the equations associated to Design III. Nevertheless, we comment briefly on the meaning of the parameters. All parameters are non-dimensional and defined identically in all three models, allowing a direct (and correct) comparison between the results. The factor *α *gives a measure of how much stronger the transcription from the activator-bound promoter is, compared to the basal transcription from the free one. The parameters *β *and *γ *contain the total non-dimensional strength of the transcription and translation for the activator and repressor, respectively. The *σ *parameter contains the DNA-binding properties of the repressor multimer scaled to the activator's. For Design II, the parameter *σ*' differs in expression from that of *σ *in the other models, justifying the different notation (see [18]). For the current study and in order to restrict to fewer parameters, we shall consider *σ *= 1, as [18]. A parameter that plays a crucial role in the existence of oscillations in the models defined above is the quotient of degradation rates Δ = *δ*_*A *_/*δ*_*R*_, with *δ*_*A*_, the activator's degradation rate. By scaling the time to the repressor's degradation rate, *δ*_*R*_, the period of the oscillations resulting from these models is expressed here in units of *δ*_*R*_, a detail that needs to be remembered when comparing the resulting periods of the different designs.

**Table 2:** The list of symbols employed in the ordinary differential equations associated to the designs from Figure 2.

Symbol	Definition & Comments
*A*	concentration of the activator (nM)
*R*	concentration of the repressor (nM)
*t*	model time (hours)
	DNA-binding constant of the activator *A*
	DNA-binding constant of the repressor *R*
	dimerization constant of the activator *A *to *A*_2_
	dimerization constant of the repressor *R *to *R*_2_
	multimerization constant of the activator *A *to *A*_*n*_
	dimerization constant of the repressor *R *to *R*_*m*_
*δ*_*A*_	degradation rate of the activator *A*
*δ*_*R*_	degradation rate of the repressor *R*
Δ	*δ*_*A*_/*δ*_*R*, _non-dimensional parameter of the model
*x*	, the nondimensional variable associated to the activator, when the activator is a dimer
*y*	, the nondimensional variable associated to the repressor, when the repressor is a dimer
*τ*	t*δ*_*R*_, the nondimensional time variable of the model
*α*	the transcriptional synergy conferred by the activator bound to DNA
*β*	, the nondimensional parameter of the transcriptional response from the activator promoter, with *γ*_*A*_, the translation rate of the activator A; *β*_*A*_, the transcription rate of the mRNA of the activator; *α*_*A*_, activator mRNA degradation; , total activator promoter number. The root order n denotes the multimerization degree of the TF binding to DNA (i.e. *n *= 2, dimer; *n *= 4, tetramer). The subscript from , the equilibrium constant for multimer formation, also denotes its dependence on the multimerization of the binding factor.
*γ*	, the nondimensional parameter of the transcriptional response from the repressor promoter, similar to *β*. See also [18]. Notice also the multimerization degree appears here as m with similar consequences as for the *β *parameter.
*σ*	, the binding ratio of the repressor compared to the activator.

In addition to the above discussion of the parameters and their meaning, let us comment briefly on the similarities and differences on the equations describing the three designs. All designs share the same rate law for the repressor, while the definitory features concern the rate law for the activator. All rate-law terms have a form of the type: production term minus degradation term. Even from the equations, one can notice that Design II is different from the other two by its particular degradation term, the post-transcriptional regulation. On the other hand, the production terms for Designs I and III differ in a subtle manner: while the transcription factors appear summed in the denominator of Design I, they appear multiplied for Design III. The *ad hoc *general rule cited in the literature for the case of more proteins affecting a gene is that the functions appear multiplied [59]. By our reasoning, the multiplication is appropriate only in cases of non-competitive transcriptional regulation. Even though cases of explicit competitive regulation are mentioned in the literature [10], the current study is the first to inquire on the differences in the dynamical behavior between the two scenarios.

**Figure 3:**
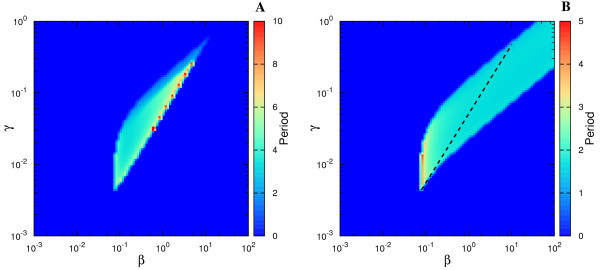
**Oscillatory features of the competition and non-competition designs associated to the studied circuit architecture**. The parameter space (*β, γ*) and the values of the oscillation period for (A) Design I and (B) Design III. The color code represents the period of oscillations depending on the (*β, γ*) pair, with *α *= 50, Δ = 10, *σ *= 1. Dark blue implies no *sustained *oscillations. As defined in the main text, the period in the color bar is expressed in units of *δ*_*R*_, the degradation rate of the repressor.

We developed numerical algorithms to simulate the equations characterizing Design I and Design III from Figure 2 and these codes are included in Additional File 2. From the resultant time series, we determined the oscillation period (if any) and represented it in Figure 3. The figures plot the value of the period for different values of *β *(abscissa) and *γ *(ordinate), where the period value is represented by a color from the color code in the right-hand-side of the figure. The dark-blue color is associated to cases lacking *sustained *oscillations.

We have chosen fixed values for Δ, *α *and *σ*, and varied *β *and *γ*, in order to compare to existing results [18]. By this study, it results that Design III is characterized by a more extended oscillatory region in the parameter space than Design I. Moreover, for Design III, the oscillatory region continues beyond the parameter limits from Figure 3B (see Additional File 3). For completion, we illustrate in Additional File 4 the change in the position of the oscillatory region when Δ (the quotient of degradation rates) is increased. For the same purpose, Additional File 5 contains the oscillatory region for Design II from [18], directly comparable with Figure 3.

By fixing Δ and allowing *β *and *γ *to vary, we may relate to the experimental context. More precisely, the binding constants  and  could in principle be experimentally-tunable as in the implementation of the oscillator from Stricker et al. (2008) [7], allowing *β *and *γ *to take different values within a certain interval. These parameters could also be varied by varying the total promoter concentration,  and  and in the expression of *β *and *γ*, respectively (Table 2). The degradation rates of the proteins could also be varied to a certain measure, and thus Δ parameter could be used as control parameter too.

**Figure 4:**
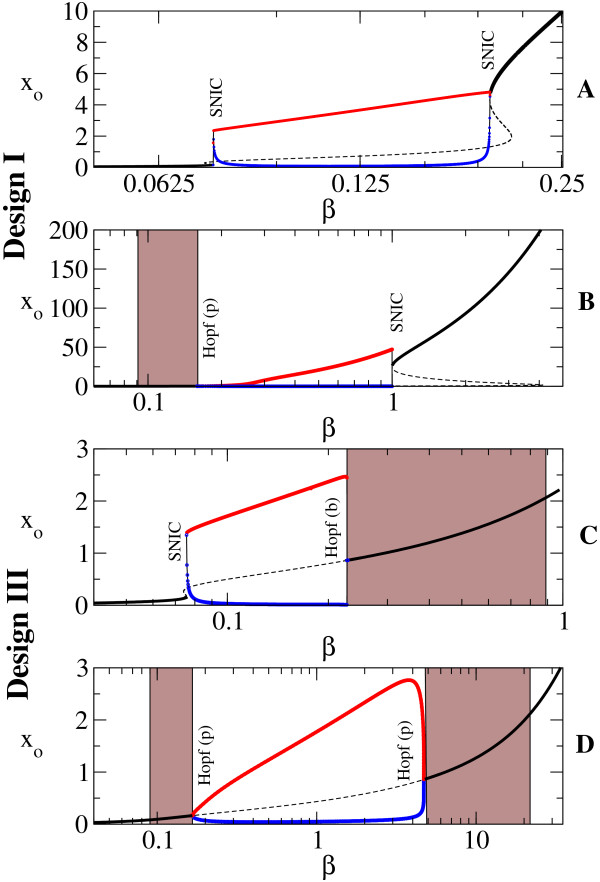
**The bifurcation diagram with *β *as control parameter**. The bifurcation diagram obtained by varying *β*, with *α *= 50 and Δ = 10, (A) and (C) *γ *= 0.01 and (B) and (D) *γ *= 0.05. It corresponds to the crossing of Figures 3A and 3B at constant *γ*. In black, we plot the stable (solid) and unstable (dashed) fixed points, *x*_*o*_, and in red and blue, the maximum and minimum, respectively, of the stable limit cycle. Notice the change in the way oscillations are triggered depending on the fixed parameters. Here Hopf(b) and Hopf(p) indicate subcritical and supercritical bifurcation, respectively. Gray areas represent damped oscillations.

In addition to the existence of the sustained oscillations, we also identified the bifurcation types that allowed this behavior. We illustrate in Figure 4 how the sustained oscillations originate when crossing the parameters space in Figures 3A and 3B from left to right (from lower to higher *β*) for two fixed values of *γ*: *γ *= 0:01 (panels A and C) and *γ *= 0:05 (panels B and D). As already identified by the studies mentioned above, two main bifurcation types are observed: the Hopf bifurcation and the saddle-node on an invariant cycle, or SNIC, bifurcation [65]. Below, we shall comment on the defining features of these two distinct types of bifurcations, without entering in excessive technical details.

As with many other bifurcation types, the Hopf bifurcation comes in two flavors: the *supercritical *(or soft) and the *subcritical *(or hard) (defined below). In both cases, the real part of the complex eigenvalues becomes positive, and the unique stable state of the system loses stability. For the supercritical type (referred to as Hopf(p) in the figures), the oscillations begin with low amplitude near the bifurcation, which increases with the change in the parameter. It is the case of Figure 4B. Even though it has the same eigenvalue behavior, the subcritical case (referred to as Hopf(b) in the figures) is more dramatic: the *sustained *oscillations start or end with high amplitude at the bifurcation point. It is a consequence of two limit cycles, a stable and an unstable one, colliding at the bifurcation point, leaving an unstable fixed point surrounded by a stable limit cycle (Figure 4C; Additional File 6).

We wish to especially stress the fact that systems undergoing Hopf bifurcations, both subcritical and supercritical, always exhibit *damped *oscillations prior to the bifurcation point (where sustained oscillations begin), whereas systems near saddle-node bifurcations do not (page 13. of Ref. [67]). One would observe the following: at a value of the control parameter that leads to negative real eigenvalues, the system stabilizes to a fixed value, without any oscillations; by changing the control parameter until the eigenvalues become complex but still with negative real part, the system would still stabilize to a fixed value, but through damped oscillatory trajectories; finally, a bifurcation is said to occur when, by further changing the control parameter, the system does not stabilize to a fixed value, but continues to oscillate indefinitely, and thus a new qualitative behavior appears. Additional File 7 illustrates an example of how sustained oscillations exist for a certain value of the control parameter Δ after the Hopf bifurcation (Δ = 4.5), and how these are preceded (that is, for values of the control parameter prior to the birth of the stable periodic orbit, Δ = 4.3) by damped oscillations.

In Figure 4, the gray areas denote ranges of *β *for which damped oscillations exist. In these ranges, the eigenvalues are complex, but with negative real part. The existence of an imaginary part of the eigenvalues leads to the oscillatory behavior, but due to the real part being negative, these oscillations are damped.

For example, damped oscillations exist for *β *>*β*_*c *_= 0.225 in Figure 4C, with a higher damping rate the farther away from the bifurcation point.

In the SNIC bifurcation, a single real eigenvalue changes sign; geometrically, a stable node and a saddle point meet, annihilate each other, and leave in their wake a limit cycle. When crossing a SNIC bifurcation, the oscillations start with very long period, and tend to stabilize to a constant value as the parameter is changed further (see also [18]). This behavior is contrasted by the Hopf bifurcation for which the oscillations have always a characteristic period. With these comments in mind, the types of bifurcations can be easily identified in Figure 3 through the color code. For Design III, the frontier around *β *≈ 0.1 is characterized by a SNIC bifurcation as *β *is increased, as the region shows long periods (Figure 4C). Crossing the oscillatory region for higher *γ*, the period appears to be constant, as both entering and leaving the oscillatory band occur through Hopf bifurcations (Figure 4D). On the other hand, for Design I, the exit from the oscillatory band occurs always through a SNIC bifurcation.

**Figure 5:**
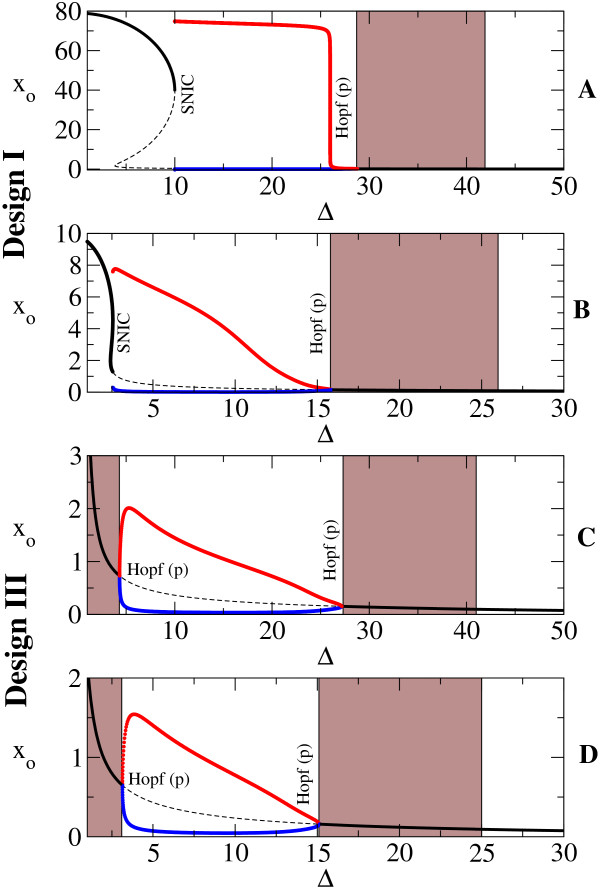
**The bifurcation diagram with Δ as control parameter. Similar to Figure 4, but having Δ as control parameter**. The fixed parameters are *α *= 50, and *β *= 1.58, *γ *= 0.079 for (A) and (C), and *α *= 50, and *β *= 0.2, *γ *= 0.04 for (B) and (D).

**Figure 6:**
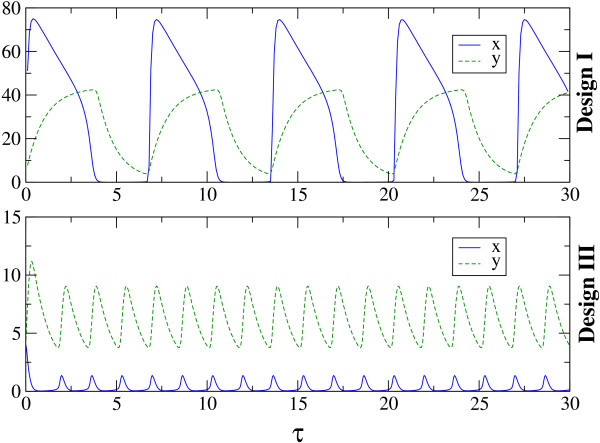
**An example of time series for the two studied designs**. Time series for Design I and Design III for the case *α *= 50, Δ = 11, *β *= 1.58, *γ *= 0.079. Notice the pulse-like oscillation of Design I compared to a more sinusoidal one for Design III, as a consequence of the type of bifurcation they originated from (Figure 5A and 5C).

As discussed by Conrad et al. [19], the parameters determine the type of bifurcations the system experiences, and we illustrate this remark by varying parameters other than *β *and *γ*. In Figure 5 we show the types of bifurcations occurring when Δ is varied for fixed *β*, *γ *. This analysis directly compares with the previous studies [18,19]. As commented above, the spiking-like oscillations are a consequence of SNIC bifurcation, while Hopf one produces more sinusoidal oscillations. An example of these two types of oscillations is illustrated in Figure 6.

Returning to Figure 4, notice also how the behavior of Design III from panel *C *matches the behavior of Design II from [19](their Fig. 3b): depending on the parameter to be varied, a design can present both types of bifurcations. Guantes&Poyatos [18] stated that Design I acts as a signal integrator (due to SNIC bifurcation) while Design II resonates with specific frequencies of the stimulus as a resonator (due to the Hopf bifurcation). Conrad et al. [19], on the other hand, highlight the fact that Design II can suffer both types of bifurcations depending on the parameters. This is the reason for using two distinct sets of parameters in Figure 4 and Figure 5, emphasizing their statement. According to Figure 4C, Design III behaves very similarly to Design II (see study of Design II in Ref. [18]) in spite of very different underlying biochemical mechanisms that produce the negative feedback. Thus we would suggest an intermediate position that states that indeed the parameters determine the type of bifurcations that occur, but only when the *biochemical mechanism *(or the resulting mathematical formulation) allows these bifurcations to occur at all. For example, Design I cannot pass from an integrator to a resonator by changing one parameter as happens for Design II and Design III.

As a final remark and returning to the discussion on the Atkinson oscillator, it is interesting to notice that the experimental implementation showed damped oscillations. We have mentioned that damped oscillations occur prior to a Hopf bifurcation, and not to SNIC bifurcation. Judging from the discussion of the two designs (Figure 4), it is reasonable to infer that the experimental implementation from [6] might be closer to Design III than to Design I. In other words, the activation and repression DNA loops might not be completely exclusive, as Atkinson et al. claim. This would be in accordance to the model studied by [44]. Nevertheless, the clarification of this issue needs further experimental studies.

#### Dynamical considerations

Apart from the computation of the parameters leading to oscillations, let us now present a dynamical perspective on the differences between Design I and III. For a better understanding of the oscillatory features of the systems, we have studied in more detail the stability properties of the fixed points associated to the three designs. More specifically, we have calculated the eigenvalues for the fixed points associated to the three designs for various parameters spaces. The Additional File 8 includes the results of this study of which we mention here only the main conclusions. We present the stability properties of the fixed point of the system for the parameters space (γ/β, σ) for fixed Δ (Figure S8.1), (γ/β, Δ) for fixed σ (Figure S8.2) and also, for comparison with Figure 3, the space (γ, β) for fixed Δ and σ (Figure S8.4). A first conclusion is that Design III shows oscillations, *both *damped and sustained, for a larger fraction of the parameter space (Figure S8.1 and S8.2), further reinforcing Figure 3. From this perspective, one could say that Design III is substantially more robust than Design I to changes in the parameters' values. Secondly, while Design III appears to be an oscillator of a specific frequency for a wide parameters range (Figure S8.5), Design I presents a wide distribution of possible oscillation periods depending on the parameters' values, due to the SNIC bifurcation. In this aspect, Design II comes as an intermediate step between the other two designs.

It is interesting to mention that, while the repressor's amplitude for the three models is similar, the distribution of activator's amplitude is specific for each design. It ranges from the queue of high amplitudes for the spiky oscillations of Design I, to an almost uniform and narrow distribution for Design III (Figure S8.5). The difference in this aspect between Design I and III is somehow expected, as the amplitude of the latter is further restrained by the term *σx*^2^*y*^2 ^at the denominator. Both Design II and III show an interesting cut-off in activator's amplitude limiting it at low values. From these features can be seen that the biochemical mechanism leaves a characteristic fingerprint on the nature of the resultant oscillations. Having observed these features in amplitude and period, we have inquired on the dynamical causes responsible for these limitations. As already illustrated by [18] in their comparison between Design I and II, the answer to this question could be found by studying the nullclines associated to the systems. The nullclines denoted as *y*_*x *_and *y*_*y *_represent the function *y*(*x*) obtained from the conditions  = 0 and  = 0, respectively. The fixed points - stable or unstable - of the system are found at the intersection of *y*_*x *_and *y*_*y*_, and the form of these functions also gives clues on the general dynamics of the system beyond the steady states. The definition of the nullclines and the extended results of this analysis are included in Additional File 9. In spite of very different  function for the three systems, it is surprising to find that *y*_*x *_for Design II and III are tightly related, with the first being the square of the other. This "similarity" however does not imply necessarily a similarity in dynamic behavior. The analysis of the nullclines as detailed in Additional File 9 shows that a change in *β *does not imply a change in the shape of the nullclines, as the shape is mainly controlled by *α*. For this reason, these two designs present a characteristic frequency and a limitation in *x *amplitude. Moreover, from the equations can be seen that, starting from an oscillation-producing parameter case, an increase in *β *implies upwards displacement of *y*_*x , *_and an increase in Δ or *γ *implies upwards displacement of *y*_*y*_. For this reason, by increasing both *β *and *γ *simultaneously, their crossing is maintained and thus the oscillations too. This does not occur for Design I for which a change in *β *implies not only a displacement, but a change in shape, leading to a three-crossing case: no oscillations. Besides explaining the loss of oscillation capability for this design beyond a certain threshold value in *β*, the shape of the nullclines, as discussed in the additional file, is responsible for the high amplitudes in both *x *and *y*, as can be see in Figure S8.5.

#### Influence of internal noise

There has been increasing acknowledgment of the fact that internal noise at cellular level - low number of molecules [68-70] - can in principle change qualitatively the behavior of the system with respect to the one predicted from noise-free (deterministic) modeling: noise-induced stabilization [71], noise-induced oscillations [29]. Moreover, there is clear evidence that the transcription and translation level are indeed noise-prone processes [72]. For these reasons, we consider that the influence of internal noise in the three systems studied here constitutes a work on its own. Nevertheless, we have chosen to mention here how noise affects the behavior of the three oscillators close to the bifurcation regime. It is observed that, for values of the parameters leading to stable states in the deterministic cases close to the bifurcation, the noise induces oscillations in all three designs, but with different characteristics (Additional File 10). While Design I and II show period distribution typical for noise-induced oscillations - long tail distribution of periods-, Design III still maintains its characteristic period. These results as well as the deterministic ones from Figure S8.5 (amplitude distribution) show that while Design I has high amplitudes, it is unreliable at period-level (long tail when noise exists), Design III has low amplitude, but it is reliable in terms of period constancy. We remind the reader that Design III is characterized also by a large fraction of parameters space leading to oscillations. All these reasons considered, Design III appears as a more reliable oscillator.

#### Other two-component oscillators

Once more, we wish to emphasize an important ingredient that makes oscillations possible in the above models: the time scale difference between the degradation rates of the two TFs given by Δ (the quotient of degradation rates). We have seen that for all these models, no oscillations are possible unless a substantial difference between the two degradation rates exists (Δ ≫ 1). The requirement thus says that *δ*_*A *_≫*δ*_*R*_. As commented also by Atkinson et al. [6], strategies for generating oscillations include increasing *δ*_*A *_and/or decreasing *δ*_*R*_. However, decreasing *δ*_*R *_is not a good strategy as we have seen that the resulting oscillation period depends on *δ*_*R*_. The shorter *δ*_*R*_, the longer the period. For example, a value of *δ*_*R *_= 0.02 *h*^-1 ^typical value of *δ*_*A *_= 1 *h*^-1 ^would result in a good Δ for the occurrence of oscillations, but in a period of about 100 hours for Design III. Such a long period, much longer than the cell-cycle period, could be altered by the intrinsic cellular division period. On the other hand, Elowitz&Leibler [4] follow the strategy of increasing *δ*_*A*_, that is synthetically design proteins that have a short life time (of the order of minutes). This strategy appears to be widespread nowadays.

Another strategy to facilitate oscillatory conditions is a more pronounced nonlinearity of the regulatory function, that is consider a Hill function of higher exponent. The mathematical model for Design III considered dimers as active TFs and one binding site for each feedback. For example, let us assume the general case for Design III. By the term *general *we refer to a non-competitive design similar to Design III, but having several bindings sites for activators and repressors. In this case thus, an approximate model would employ the general form of Hill function:(1)(2)

**Figure 7:**
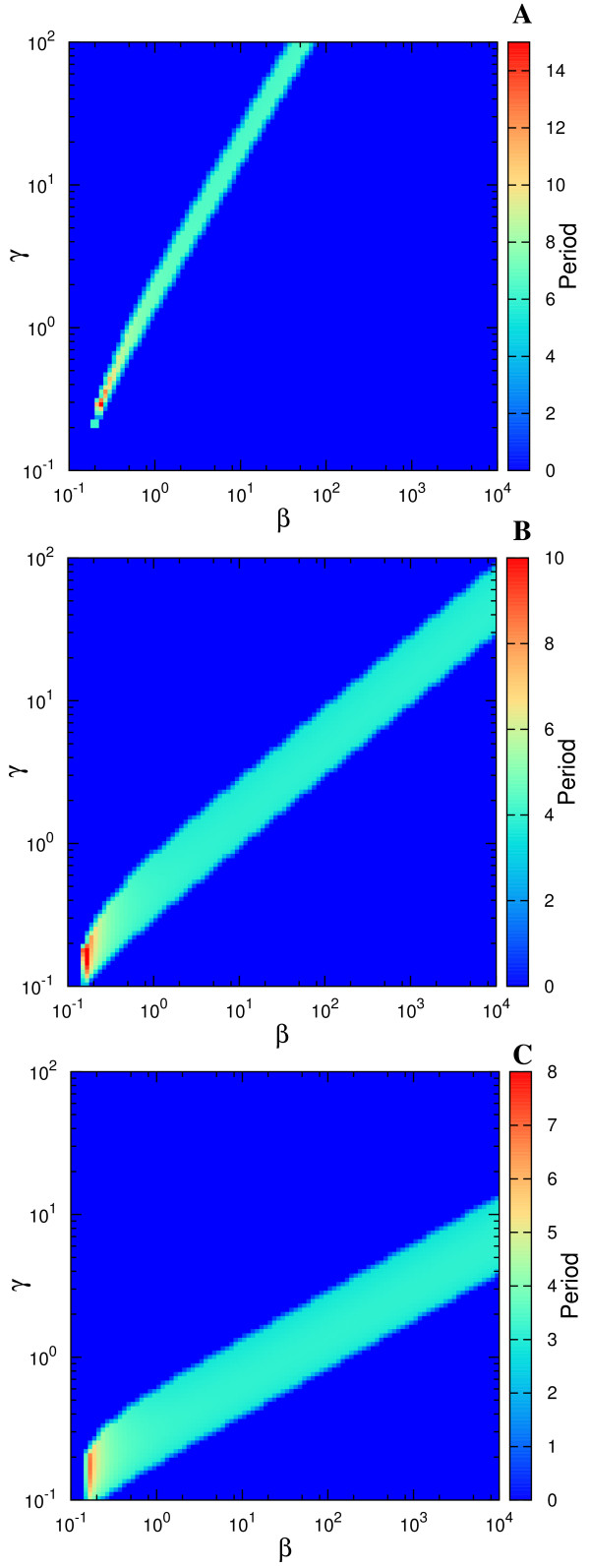
**Oscillatory features of the simplified Atkinson model**. The behavior of the simplified model of the Atkinson oscillator from eqs. (1-2) for *α *= 50, Δ = 1, *n *= 3 and (A) *m *= 1,(B) *m *= 2 and (C) *m *= 3.

However, keep in mind that the parameters *β *and *γ *depend on the degree of multimerization of the TFs. If *n *≠ 2 and *m *≠ 2, the parameters *β *and *γ *differ in expression from those employed in the previous section, and thus a direct comparison with the previous results is difficult. Nevertheless, the interesting remark to make here is that oscillations are possible even for Δ = 1 (no difference in degradation rates) when *n *= 3, as seen in Figure 7. The figures illustrate the general behavior of the model to changes in the multimerization degree of the repressor. It is apparent from these figures that *n*, the nonlinearity of the positive feedback, is responsible for the *existence *of the oscillations, while *m *relates to the extension of the parameter space leading to these oscillations: the higher the *m *(degree of repressor's multimerization), the wider the oscillatory region.

**Figure 8:**
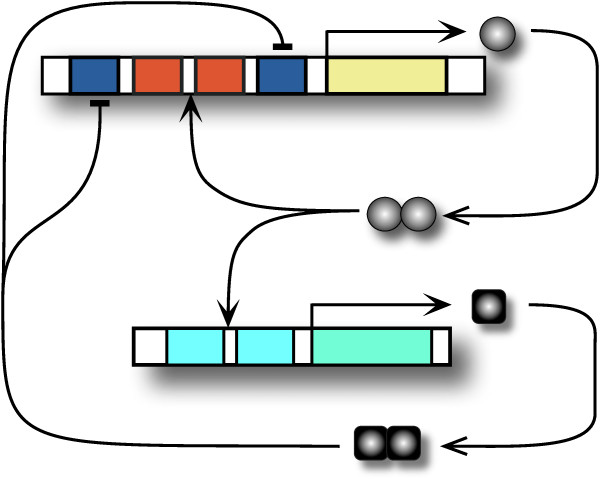
**The scheme of the experimental design from **[6].

**Figure 9:**
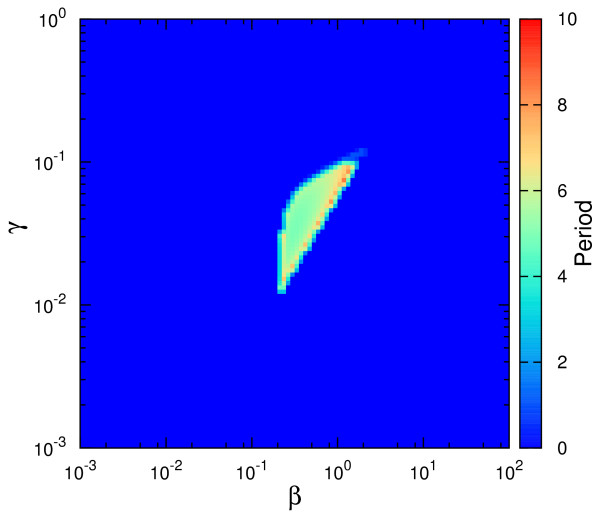
**Oscillatory features of the Atkinson model**. The numerical simulations for the corresponding model in eqs. (3-4) and Figure 8 with Δ = 10 and *α *= 50. When compared to Figure 3A, it is visible that the oscillatory region is significantly reduced.

Again concerning the nonlinearity of the feedback, let us return to the experimental implementation of the Atkinson oscillator. The experimental design [6] appears in Figure 8 (and also schematically in Figure 1C), including two binding sites for each TF. The full-competition scenario occurs under the hypothesis that when both sites of the activator (or of the repressor) are occupied by the respective dimers, a DNA loop is formed, blocking the formation of the repressor's loop (or of the activator's loop, respectively). The mathematical model describing this scenario would be based on Design I but characterized by *n *= *m *= 4:(3)(4)

and where Δ, *β *and *γ *have the same expression as in Design I and thus are directly comparable. Notice in Figure 9 the reduced sustained-oscillations region for the model in eqs. (3-4) compared to Figure 3A. It is interesting that, in this case, the increase in the nonlinearity of the feedbacks leads to a decrease of the oscillatory region.

The model from eqs. (1-2) is a simplified version of the Atkinson model as described by Scott et al. [44]. There, the model included the approximation of the Hill functions as above, but considered explicitly the dynamics of the messenger RNAs, resulting in a system of 4 variables instead of 2 variables as employed here. The approximation that allowed us to consider only the protein variables, as in the above equations, is based on the fact that translation is much faster than transcription, and thus as soon as the mRNA is formed, one can consider that the subsequent protein is formed too. It is a drastic approximation and it has already been proved to have relevant consequences: explicitly modeling the mRNAs yields a better fit to the experimental data than the simplified model [7]. Nevertheless, the simplified model is a more tractable tool for studying the general characteristics of the system.

Still in two-gene systems, another strategy to facilitate oscillations is introducing another positive feedback for Design I, more precisely an autocatalysis on the repressor. This eliminates the necessity of large Δ, while maintaining dimers and one binding site. Of course, this leads to another architecture than the one shown in Figure 2A. This architecture is contemplated by [46] (see Table 1 and Figure 1G):(5)(6)

**Figure 10:**
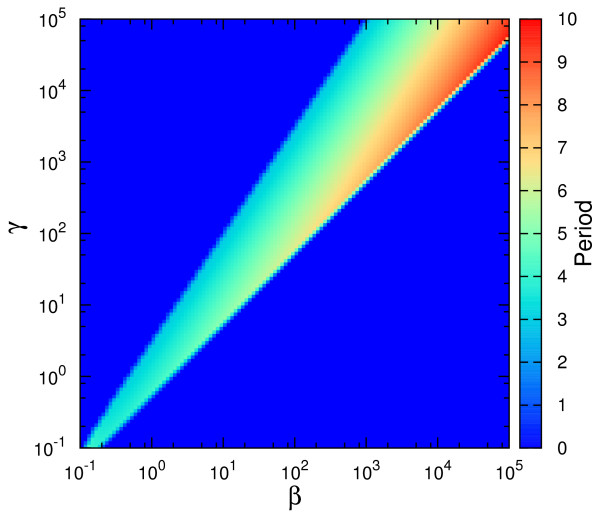
**Oscillatory features of the circuit introduced by Smolen et al**. [46]. The behavior of the oscillator from eqs. (5-6) introduced in [46] with *α *= 50, Δ = 4, *σ *= 1. The scale differs from that of Figures 3 and 7, but nevertheless notice the difference in the location and extension of the oscillatory region when compared to these figures.

Notice in Figure 10 how the oscillatory region considerably changes in this architecture. The parameters are directly comparable with the Designs I, II and III. Let us notice that the activator must be a multimer (at least a dimer) for the occurrence of oscillations, but also a significant value of Δ is also needed, as Δ = 1 is not sufficient for oscillations to occur. As already discussed for the Atkinson model above, the degree of multimerization of the repressor is not as critical as that of the activator. Also Smolen et al. [46] introduce the above architecture considering the repressor as monomer, and emphasizing the necessity of a dimer activator for the oscillations to occur. Once again, the nonlinearity of the transcriptional response is the driving factor in the instability required for the oscillations.

## Conclusions

In the present work, we have analyzed two DNA-binding mechanisms in composed promoter sites and how they affect the existence of oscillations in genetic circuits. There is general consensus that both positive and negative genetic interactions are needed to obtain a robust oscillator [73]. Given a number of such interactions, there exist several mathematical and experimental proposals for oscillators. Even though there is sufficient proof that two positive interactions and a negative one would produce a genetic oscillator, there are various biochemical mechanisms compatible with such an architecture (Figure 2). The differences between these mechanisms and their consequences at the level of robustness and reliability of the resultant oscillator are a new direction of study [18,47].

From our results, we have emphasized the fact that, when these interactions, either positive or negative, are accomplished through transcriptional regulation, the interactions between various binding sites on the promoter need to be taken into consideration. We showed the non-competition scenario to present robustness to parameter changes defined by the existence of oscillatory solutions for a much wider parameters range than for the competition one. Moreover, the two scenarios imply different routes toward the occurrence of oscillations, that is different bifurcation types. While the competition model is mainly associated to the SNIC bifurcation, the non-competition one relies more on the Hopf bifurcation. While the former bifurcation type leads to more spiking-like oscillations, the latter shows more sinusoidal ones. In addition, a characteristic of the Hopf bifurcation is that sustained oscillations are preceded by damping oscillations. This may prove to be an important feature to consider when interpreting experimental data. Many other interesting properties arise as a consequence of the type of the oscillation-generating bifurcation, an issue that has been extensively analyzed in previous works [18,19,67] and thus has not been discussed here. However, consistent with previous works, we found that which parameter is changed - and in which range it is changed - determines the bifurcations that occur. Nevertheless, not only the parameters are responsible for the resultant dynamics of the system, as argued by Conrad et al. [19], but most important of all, it is the underlying logic of the biochemical mechanism that makes the dynamics possible at all. In addition, we conclude from our results that also very different designs (or biochemical mechanisms) can produce very similar behaviors. The non-competition scenario analyzed here produces an overall dynamic behavior similar to the post-transcriptional control scenario described by Guantes&Poyatos [18].

The Atkinson oscillator introduced and experimentally implemented by [6] has been modeled both through competition [18] and non-competition models [44]. As the experimental implementation failed to provide sustained oscillations, it is interesting to assess the implications of the two scenarios explored in the current study in the context of this experimental implementation. The goal of the current study was to pinpoint the consequences of the biochemical mechanisms and the mathematical approximations taken into consideration when constructing the associated theoretical model, and thus not to present a detailed study of the already-implemented experimental design [6]. Nevertheless, we consider that our work sheds new light on the behavior of this genetic oscillator.

Here we have used simplified models of genetic circuits. In this simplification, the modeling of genetic circuits includes only the temporal evolution of the protein concentration [45,74]. This allows a straight-forward mathematical analysis of the system and its properties. Moreover, we have chosen here to employ the simplified approach as our goal was not a characterization of a specific experimental design, but to emphasize the importance of taking into consideration the underlying biochemical mechanisms and the promoter architecture at the moment of constructing a mathematical model of a genetic circuit. In summary, to have the largest chances of obtaining oscillations, synthetic biologists should build circuits of the form of Design III, avoiding competition between activator and repressor. Additionally, faster degradation rates for activators than repressors are preferred (or adding an autocatalysis for the repressor). Finally, achieving non-linear transcription responses (multimeric factors, at least dimers, and/or multiple binding sites) also favor oscillatory behaviour. Models that are more robust to parameter changes should be easier to construct experimentally. We therefore hope that this work will help bring closer the theoretical and experimental communities in the fields of systems and synthetic biology.

## Methods

For the numerical simulations, we developed C++ codes employing routines from the free-software GNU Scientific Library (Additional File 2). In addition, the bifurcation diagrams employ results obtained with the use of the AUTO software within XPPAUT program.

## Authors' contributions

All authors contributed to the design and coordination of the study. AM performed the computational implementations and prepared the original draft, which was revised by RVS, and subsequently by MC and MI. All authors read and approved the final manuscript.

## Supplementary Material

Additional file 1 **Transcriptional regulation and model description**. A PDF file containing a brief introduction to transcriptional regulation and the terminology employed in the main text. The details necessary for obtaining the equations employed in this study are also included here. Click here for file

Additional file 2 The C++ codes developed for the numerical simulations, as well as the instructions of their use. Click here for file

Additional file 3 Oscillatory region for an extended parameter space for Design III. Click here for file

Additional file 4 Comparison between Design I and Design III for Δ = 20. Click here for file

Additional file 5 **The behavior of Design II from Guantes&Poyatos**[18]. Click here for file

Additional file 6 Example of a subcritical Hopf bifurcation. Click here for file

Additional file 7 The trajectory behavior prior to a Hopf and to a SNIC bifurcation. Click here for file

Additional file 8 Robustness to parameter changes. Click here for file

Additional file 9 Dynamical differences between the designs: discussion of the nullclines. Click here for file

Additional file 10 The influence of internal noise. Click here for file
