# Supplementary material for: Avoiding transcription factor competition at promoter level increases the chances of obtaining oscillation
Source: BMC Syst Biol. 2010 May 17;4:66. doi: 10.1186/1752-0509-4-66 (PMC2898670; doi:10.1186/1752-0509-4-66)
Supplement: Additional file 1 — Transcriptional regulation and model description. A PDF file containing a brief introduction to transcriptional regulation and the terminology employed in the main text. The details necessary for obtaining the equations employed in this study are also included here. [file 1752-0509-4-66-S1.PDF]

Additional File 1 of *Avoiding transcription factor competition at promoter level increases the chances of obtaining oscillations*

Transcriptional regulation

Stripping gene expression down to bare bones, one can say that two phases are common to all organisms: the gene is transcribed into messenger RNA (mRNA) and this mRNA is then translated into protein. Ever since the pioneering works of F. Jacob and J. Monod in the 1950s, the study of transcriptional regulation in prokaryotes and eukaryotes has revealed many other crucial actors in this elaborated play [1]. We shall consider here a simplified version of the process [2, 3] that allows us a direct relation to the Hill function, the one generally employed in mathematical modeling of genetic circuits. The Hill function accounts for the cooperative nature (positive or negative) of various binding sites in the promoter region. It provides a simple way of introducing this cooperative effect, even though it may not be an accurate representation of the underlying dynamics. But let us consider a simple case of transcriptional regulation by a protein that acts as regulator in the dimer form. Let X, X_2, D denote the protein, the associated dimer and a (free) DNA promoter site, respectively. We may write the equilibrium reactions

$$X + X \overset{K_d}{\longleftrightarrow} X_2 \tag{1}$$

$$D + X_2 \overset{K_b}{\longleftrightarrow} D_b \tag{2}$$

with D_b denoting the promoter state occupied with the dimer and K_i, the equilibrium constants. Defining concentrations as $x = [X], x_2 = [X_2], d_f = [D], d_b = [D_b]$, one has: $x_2 = K_d x^2$, $d_b = K_b d_f x_2 = K_b K_d d_f x^2$, and we define $K \equiv K_b K_d$. Moreover, the total concentration of the promoter, d_T, is constant: $d_f + d_b = d_T$, leading to the expressions for the *free* DNA promoter $d_f = d_T \frac{1}{1+Kx^2}$ and occupied or *bound* promoter $d_b = d_T \frac{Kx^2}{1+Kx^2}$. The rate of transcription, r, that is the rate of mRNA production will depend on whether the transcription occurs predominantly from d_f or from d_b:

$$\begin{aligned} r &= \alpha_f d_f + \alpha_b d_b \\ &= d_T \frac{\alpha_f + \alpha_b K x^2}{1 + Kx^2} \\ &= \begin{cases} b + d_T \alpha_b \ \frac{Kx^n}{1+Kx^n} & \text{if } \mathrm{x_n} \text{ is an activator } (\alpha_\mathrm{b} \gg \alpha_\mathrm{f}) \\ b + d_T \alpha_f \ \frac{1}{1+Kx^n} & \text{if } \mathrm{x_n} \text{ is an inhibitor } (\alpha_\mathrm{b} \ll \alpha_\mathrm{f}) \end{cases} \end{aligned} \tag{3}$$

where the parameter α_b represents the degree to which the transcription rate is modified when the dimer is bound to the promoter. It is referred to also as transcriptional synergy [4]. We have employed the general Hill exponent n, with the case above being characterized by $n = 2$. The parameter b is defined by $b \equiv d_T |\alpha_b - \alpha_f|$.

In the case of the dimer on a binding site ($n = 2$) described above, one can say that the Hill function is an exact representation of the system. This comes to say that the approximations employed to obtain this function rely exclusively on the equilibrium conditions (eqs. 1, 2). No other approximations are used. The generalization to any other exponent n is employed when the promoter contains several binding sites, each characterized by its binding constants, K_{bi}, and degrees of transcription,α_i, and which are averaged into a simple function as in eqs. (3). The higher the exponent n, the closer the Hill function to an OFF-ON or step-like process. In Figure 1 we illustrate the consequences of increasing the exponent n, in other words, the ultrasensitivity of the response or cooperativity. Identifying chemical mechanisms that lead to ultrasensitive response has been a priority in both fundamental and synthetic biology [5–7]. However, high exponents (e.g. $n \gg 4$) are difficult to obtain through the DNA-binding mechanisms that we comment in the current study. From the mathematical point of view, only a few studies provide clarifying discussions on the modeling of binding cooperativity and transcriptional synergy [4, 8].

The models

The Supporting information file of [9] (GP-S, in short) includes a detailed description of how the equations corresponding to Design I and II were built. For completion, we provide here the similar details associated to Design III. The reactions for Design III are completed by adding a few reactions to those detailed in GP-S associated to Design I (their reactions 1,2,3,4) and these reactions are

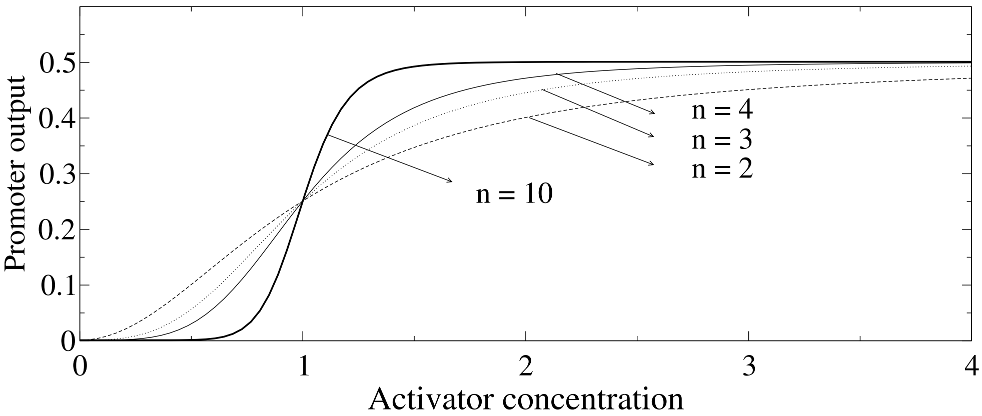

FIG. 1: Examples of an activator Hill function from eqs. (3) as a function of x, the activator concentration. The parameters are $b = 0.001$, $K = 1$, $d_T\alpha_b = 0.5$. The higher the exponent, the closer the behavior of the function to a switch.

$$P_A A_m + R_n \underset{k'_{-2}}{\overset{k'_2/\Omega}{\rightleftharpoons}} P_A A_m R_n \tag{4}$$

$$P_A R_n + A_m \underset{k'_{-3}}{\overset{k'_3/\Omega}{\rightleftharpoons}} P_A A_m R_n \tag{5}$$

$$\tag{6}$$

where the notations are: P_A, activator promoter region, A_m, activator multimer, R_n, repressor multimer, Ω, the parameter that takes into account the system size, and includes cell volume and Avogadro's number. The kinetic constants k'_2 and k'_{-2} denote the association and dissociation kinetics, respectively of the repressor when the activator is bound; and similar for k'_3 and k'_{-3}. Under these assumptions, the total activator promoter number becomes $P_A^T = P_A + P_A R_n + P_A A_m + P_A A_m R_n =$ constant. Thus compared to eqs. (5) in GP-S, only the production of m_A will change by introducing the above reaction. One can consider in a first approximation that transcription occurs only when the repressor is not bound (only from P_A and $P_A A_m$) and referring again to the notations in GP-S, the equation describing the production of m_A is:

$$\begin{aligned}
\frac{dm_A}{dt} &= \beta_A P_A + \beta_A \alpha P_A P_A A_m \\
&= \beta_A P_A (1 + \rho K_3 K_A A^m) \\
&= \beta_A P_A^T \frac{1 + \rho K_3 K_A A^m}{1 + K_2 K_R R^n + K_3 K_A A^m + (K'_2 K_3 + K_2 K'_3) K_R K_A A^m R^n} \\
&= \beta_A P_A^T \frac{1 + \rho K_3 K_A A^m}{(1 + K_2 K_R R^n + K_3 K_A A^m + \lambda K_2 K_3 K_A K_R A^m R^n)}
\end{aligned} \tag{7}$$

where we have introduced the notations $K'_2 \equiv k'_2\Omega/k'_{-2}$, $K'_3 \equiv k'_3\Omega/k'_{-3}$, $\lambda \equiv K'_2/K_2 + K'_3/K_3)$ (with the rest of notations identical to those from GP-S). In the current study, we have concentrated only on the case $\lambda = 1$. For this case, the above equation can be rewritten as

$$\frac{dm_A}{dt} = \beta_A P_A^T \frac{1 + \alpha K_3 K_A A^m}{1 + K_3 K_A A^m} \frac{1}{1 + K_2 K_R R^n}, \tag{8}$$

With the notations and approximations employed in GP-S, eq. 8 becomes

$$\frac{dx}{d\tau} = \Delta \left(\beta \frac{1 + \alpha x^m}{(1 + x^m)(1 + \sigma y^n)} - x \right), \tag{9}$$

having changed some notations with respect to those from GP-S (here, α is ρ in GP-S, and β is ξ_x in GP-S). The first term on the right-hand-side is the form encountered in some extant works [10], as it is generally considered that, when more than one protein affects a single gene, the Hill equations are multiplied [11], without considering the existence or lack of competition.

From the above equation, one can interpret the repressor as a modulator of the action of the activator by tuning the production rate through the factor $1/(1+\sigma y^n)$. This effect is more evident when considering the general case from eq. (7), where $\lambda \neq 1$ can be envisioned as the modulating parameter. Design I of complete exclusion is recovered from $\lambda = 0$ ($k'_2 = k'_3 = 0$). Another particular case is $\lambda = 2$ for which the binding of repressor and activator are completely independent ($k'_2 = k2$, $k'_3 = k3$, $k'_{-2} = k_{-2}$, $k'_{-3} = k_{-3}$). We have always considered that the binding of the repressor implies a drastic reduction in the transcription rate (the transcription occurs only if the promoter is free or bound with the activator, with all the other combinations not participating to the production rate in a significant measure). Even though this might not be completely true, and the repressor-bound promoter might lead to a low production of mRNAs, we shall consider here the case that this low production is insignificant.

---

[1] Ptashne M, Gann A: *Genes & signals.* Cold Spring Harbor Lab. Press 2002.

[2] Hasty J, Isaacs F, Dolnik M, McMillen D, Collins JJ: **Designer gene networks: Towards fundamental cellular control.** *Chaos* 2001, **11**:207–220, [http://dx.doi.org/10.1063/1.1345702].

[3] Hasty J, Dolnik M, Rottschäfer V, Collins JJ: **Synthetic gene network for entraining and amplifying cellular oscillations.** *Phys Rev Lett* 2002, **88**(14):148101.

[4] Veitia RA: **A sigmoidal transcriptional response: cooperativity, synergy and dosage effects.** *Biol Rev Camb Philos Soc* 2003, **78**:149–170.

[5] Ferrell JE, Xiong W: **Bistability in cell signaling: How to make continuous processes discontinuous, and reversible processes irreversible.** *Chaos* 2001, **11**:227–236, [http://dx.doi.org/10.1063/1.1349894].

[6] Buchler NE, Gerland U, Hwa T: **Nonlinear protein degradation and the function of genetic circuits.** *Proc Natl Acad Sci U S A* 2005, **102**(27):9559–9564, [http://dx.doi.org/10.1073/pnas.0409553102].

[7] Buchler NE, Louis M: **Molecular titration and ultrasensitivity in regulatory networks.** *J Mol Biol* 2008, **384**(5):1106–1119, [http://dx.doi.org/10.1016/j.jmb.2008.09.079].

[8] Schuster P: **Binding of activators and repressors to DNA. Part I: Equilibria**. Tech. rep., Working Paper 05-05-016, Santa Fe Institute, Santa Fe, NM 2005, [www.santafe.edu/research/publications/\\wpabstract/200505016].

[9] Guantes R, Poyatos JF: **Dynamical principles of two-component genetic oscillators.** *PLoS Comput Biol* 2006, **2**(3):e30, [http://dx.doi.org/10.1371/journal.pcbi.0020030].

[10] Scott M, Ingalls B, Kaern M: **Estimations of intrinsic and extrinsic noise in models of nonlinear genetic networks.** *Chaos* 2006, **16**(2):026107, [http://dx.doi.org/10.1063/1.2211787].

[11] Paladugu SR, Chickarmane V, Deckard A, Frumkin JP, McCormack M, Sauro HM: **In silico evolution of functional modules in biochemical networks.** *Syst Biol (Stevenage)* 2006, **153**(4):223–235.
