# Supplementary material for: Avoiding transcription factor competition at promoter level increases the chances of obtaining oscillation
Source: BMC Syst Biol. 2010 May 17;4:66. doi: 10.1186/1752-0509-4-66 (PMC2898670; doi:10.1186/1752-0509-4-66)
Supplement: Additional file 2 — The C++ codes developed for the numerical simulations, as well as the instructions of their use. [file 1752-0509-4-66-S2.ZIP › ReadmeProgramFiles.pdf]

README for Program Files

All programs require the GNU Scientific Library (GSL) that is a numerical library for C and C++ programmers. It is free software under the GNU General Public License.
http://www.gnu.org/software/gsl/

The generic compilation command for all programs is:

gcc -o *<filename>* *<filename>*.C -lm -lgsl -lgslcblas -lstdc++

From the programs provided, *<filename>* can be *DesignI* (relating to Figure 3 in the main manuscript), *DesignIII* (Figure 4; Figure,8,9,10; eqs. 1; Figure 12, eqs. 2), *Smolen* (Figure 13; eqs. 3).

The command for the use of the program is:

./*<filename>* **<alpha> <beta> <gamma> <Delta>**

The DEFAULT Hill coefficients for Design III are **n**=2 and **m**=2. However, in accordance to eq.(1) in the main manuscript, if other values for **n** and **m** are desired, the general form of Design III appears as

./*DesignIII* **<alpha> <beta> <gamma> <Delta> <n> <m>**

e.g. ./*DesignIII* **50 100 2 1 3 3**

that would correspond with an example from Figure 10. The output of these programs includes the timeseries file *<filename>*.dat (time, x, y) , and the main features of the behavior that appear written at *stdout*, such as, for the example above:

Alpha	*50*
Beta	*100*
Gamma	*2*
Delta	*1*
Oscillator?	*Yes*
Period_x	*3.04538*
VariancePeriod_x	*0.07459*
Amplitude_x	*0.808739*
VarianceAmplitude_x	*2.53174e-06*
Period_y	*3.04656*
VariancePeriod_y	*0.074045*
Amplitude_y	*19.1929*
VarianceAmplitude_y	*0.000150*
x_min	*0.238982*
x_max	*0.810261*
y_min	*6.8537*
y_max	*19.167*
