# Supplementary material for: Avoiding transcription factor competition at promoter level increases the chances of obtaining oscillation
Source: BMC Syst Biol. 2010 May 17;4:66. doi: 10.1186/1752-0509-4-66 (PMC2898670; doi:10.1186/1752-0509-4-66)
Supplement: Additional file 3 — Oscillatory region for an extended parameter space for Design III. [file 1752-0509-4-66-S3.PDF]

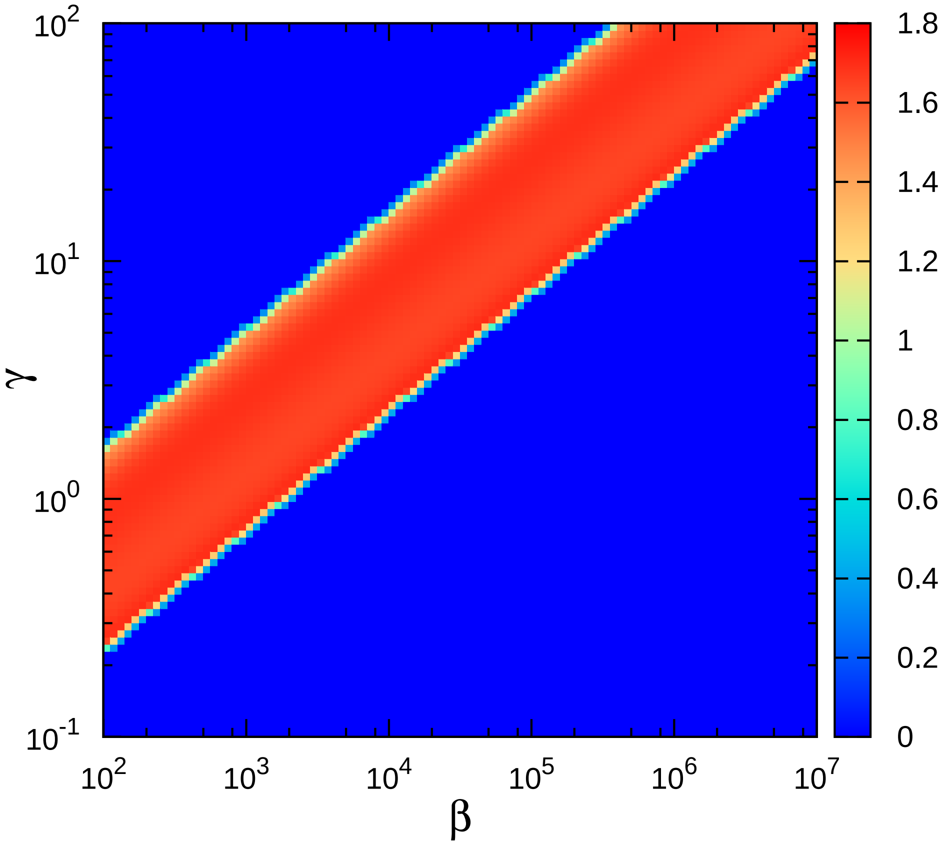

Figure S3: Similar to Figure 4 in the main text, but for an extended region of the parameter space (β, γ) for Design III. No oscillations are found for Design I in this region.
