# Supplementary material for: Avoiding transcription factor competition at promoter level increases the chances of obtaining oscillation
Source: BMC Syst Biol. 2010 May 17;4:66. doi: 10.1186/1752-0509-4-66 (PMC2898670; doi:10.1186/1752-0509-4-66)
Supplement: Additional file 4 — Comparison between Design I and Design III for Δ = 20. [file 1752-0509-4-66-S4.PDF]

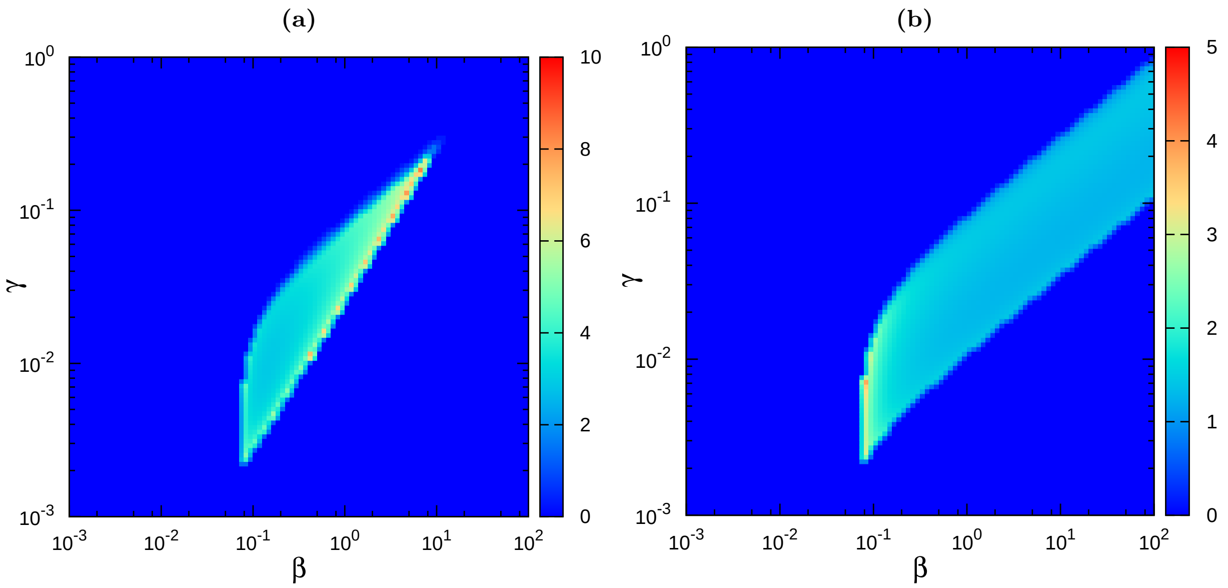

Figure S4: The comparison between Design I (left) and Design III (right) for $\Delta = 20$ compared to Figures 3 and 4 in the main text where $\Delta = 10$. The color code represents the period of oscillations depending on the (β,γ) pair, with $\alpha = 50$, $\sigma = 1$. Remember that the period is expressed in units of δ_R, the degradation rate of the repressor. Thus compared to Figures 3 and 4, changing Δ can be achieved through changing δ_R.
