# Supplementary material for: Avoiding transcription factor competition at promoter level increases the chances of obtaining oscillation
Source: BMC Syst Biol. 2010 May 17;4:66. doi: 10.1186/1752-0509-4-66 (PMC2898670; doi:10.1186/1752-0509-4-66)
Supplement: Additional file 5 — The behavior of Design II from Guantes&Poyatos[18]. [file 1752-0509-4-66-S5.PDF]

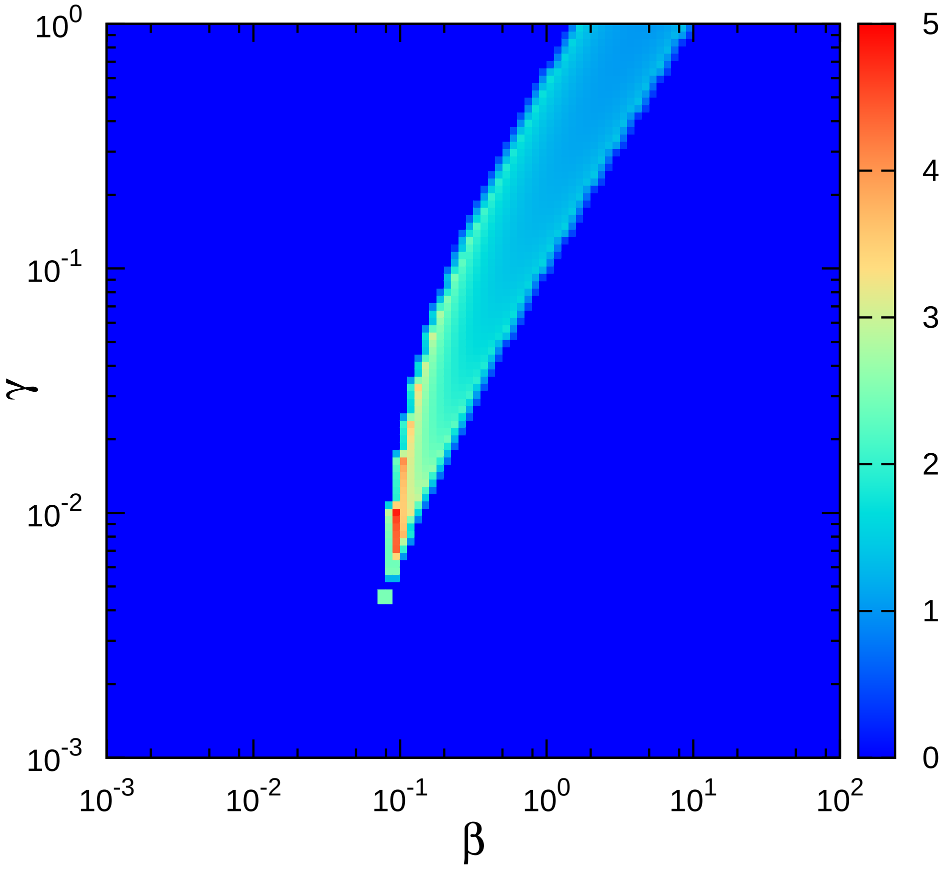

Figure S5: For comparison with Figures 3 and 4 in the main text, we illustrate here the behavior of the Design II of [1] for $\alpha = 50$, $\Delta = 10$, $\sigma' = 1$. Remember that σ for Design I and Design III has a different meaning than σ' of Design II, which makes the comparison somewhat tricky (details in the Supplementary material of [1]).

[1] Guantes R, Poyatos JF: **Dynamical principles of two-component genetic oscillators.** *PLoS Comput Biol* 2006, **2**(3):e30, [http://dx.doi.org/10.1371/journal.pcbi.0020030].
