# Supplementary material for: Avoiding transcription factor competition at promoter level increases the chances of obtaining oscillation
Source: BMC Syst Biol. 2010 May 17;4:66. doi: 10.1186/1752-0509-4-66 (PMC2898670; doi:10.1186/1752-0509-4-66)
Supplement: Additional file 6 — Example of a subcritical Hopf bifurcation. [file 1752-0509-4-66-S6.PDF]

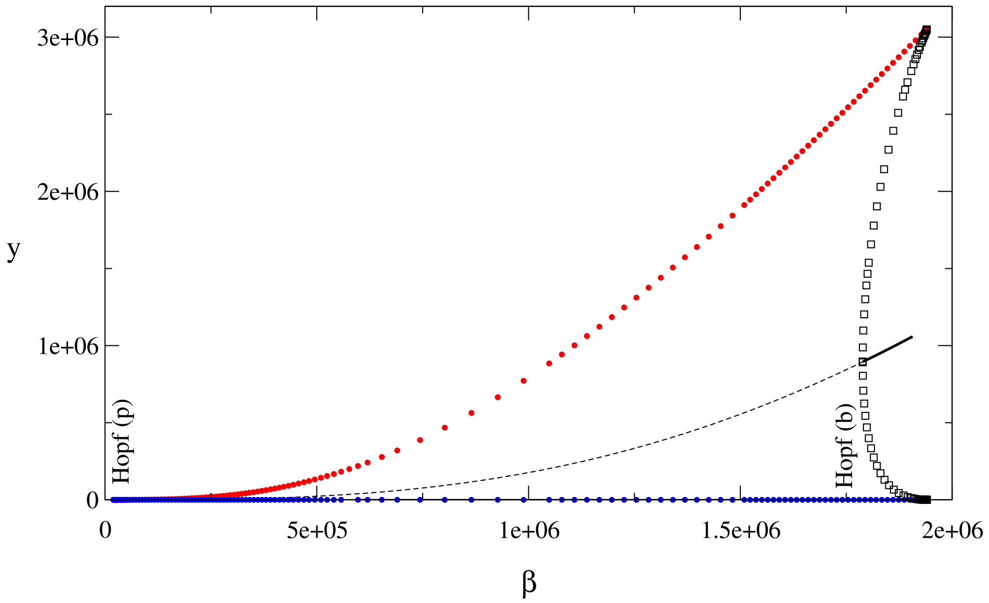

Figure S6: A detailed example of a subcritical Hopf bifurcation for the model in eqs. 3 with $\Delta = 4$, $\alpha = 3$, $\gamma = 10^6$. Filled circles denote a stable limit cycle (red: maximum , blue: minimum), while the empty circle denote the unstable limit cycle. The solid line represents stable state, while the dashed line represents the existence of an unstable fixed point. The figures in the main text illustrate only the stable limit cycle.
