# Supplementary material for: Avoiding transcription factor competition at promoter level increases the chances of obtaining oscillation
Source: BMC Syst Biol. 2010 May 17;4:66. doi: 10.1186/1752-0509-4-66 (PMC2898670; doi:10.1186/1752-0509-4-66)
Supplement: Additional file 7 — The trajectory behavior prior to a Hopf and to a SNIC bifurcation. [file 1752-0509-4-66-S7.PDF]

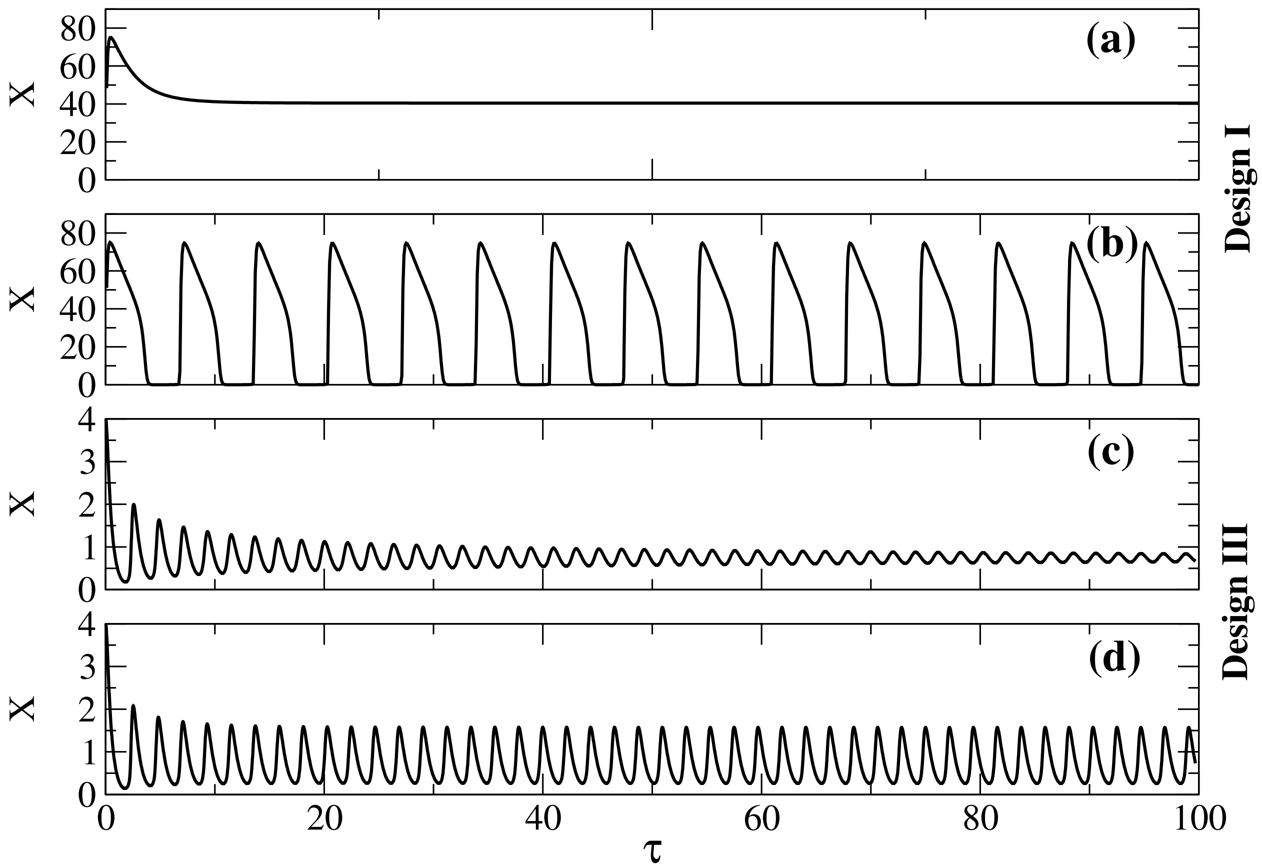

Figure S7: Damped oscillations are associated with Hopf bifurcation rather than to SNIC bifurcation. Here are represented two cases, one before (a: $\Delta = 10$ and c: $\Delta = 4.3$) and one after (b: $\Delta = 11$ and d: $\Delta = 4.5$) the bifurcation for Design I and Design III with $\alpha = 50$, $\beta = 1.58$, $\gamma = 0.079$. See Figure 6a and c for the bifurcations.
