# Supplementary material for: Avoiding transcription factor competition at promoter level increases the chances of obtaining oscillation
Source: BMC Syst Biol. 2010 May 17;4:66. doi: 10.1186/1752-0509-4-66 (PMC2898670; doi:10.1186/1752-0509-4-66)
Supplement: Additional file 8 — Robustness to parameter changes. [file 1752-0509-4-66-S8.PDF]

Additional File 8 of
Avoiding transcription factor competition at promoter level increases the chances of obtaining oscillations

Robustness to parameters

As commented in the main text, the oscillatory properties depend not only on the biological regulatory mechanism, but also on the control parameter (i.e. the parameter that is varied). Therefore, a general perspective on the oscillatory features for wider and more diverse parameter spaces than the one presented in the main text would provide a better comparison between the two models. Also, in order to compare with the results on Design II (and Design I) from [1], we shall perform similar studies and employ, in general, the values of the parameters presented in their Supporting Information File. First, we considered fixed Δ and β, and varied the parameters γ and σ. More specifically, we used $\Delta = 10$, $\beta = 1.58$, and $\sigma \in (0, 10]$ and $\gamma/\beta \in (0, 0.2]$. We remind the reader that σ is a ratio between the DNA-binding constants of the repressor compared to that of the activator, thus increasing values of σ denote higher affinity of the repressor, and thus impeded activated state, leading to a lower production of activator. On the other hand, increasing γ implies a higher production rate of the repressor.

In the figures below, we show the oscillatory features of the parameter space $(\gamma/\beta, \sigma)$ (Figure S8.1) and $(\gamma/\beta, \Delta)$ (Figure S8.2). From the definition of the colored regions in Figure S8.1, one can also infer the type of bifurcations: passing from white to black region - SNIC bifurcation; from green to red to black region - Hopf bifurcation. From this figure, it can be observed that Design III presents a wider sustained-oscillations region (black region) than Design I, with Design III avoiding the SNIC bifurcation. On the other hand, important differences are seen when comparing Design II to Design III. As discussed in the main text, the shape of the y_x nullclines of these two models are similar. From that, one might have expected similarity in the oscillatory behavior too. However, it appears that the differences are enough to affect the characteristics of the fixed point, its stability features, and by this, affect the features of the oscillations. More precisely, due to the presence of complex eigenvalue in the black regions of Design III, the oscillations are expected to be more sinusoidal, while for the blue regions in Design II, they should be more spike-like (real eigenvalue). We have chosen an example to illustrate these features and it appears in Figure S8.3.

From the results in Figure S8.2, one can see that the conclusions drawn from Figure S8.1 stand also for the parameter space $(\gamma/\beta, \Delta)$. We remind the reader that the parameter Δ is crucial for the existence of oscillations in all three models, as high values of Δ are required for sustained oscillations. Otherwise, more binding sites for the activator are needed such that cooperativity could achieve a higher nonlinearity (higher Hill exponent). However, a high value of $\Delta \equiv \delta_A/\Delta_R$ implies a low value of δ_R, thus a long half-life for the repressor and thus longer periods. Short periods are prefered from the experimental point of view, and thus it is prefered that Δ has low values. This can be achieved easier for Design III for which, moreover, damped oscillations are easier to encounter too. As discussed in [1], Design II requires higher Δ values than Design I to produce oscillations, but they persist in a wider fraction of the parameter space.

In addition, for all designs and both Figures S8.1 and S8.2, as expected for relaxation-based oscillators, the ratio γ/β must be small in order to obtain oscillations, and thus the oscillations are quenched when this ratio increases, independently on the value of the other parameters.

In order to relate better to the main manuscript, we have also represented Figures S8.1 and S8.2 in a similar way to Figure 3 from the main text. Notice also the extension of the damped-oscillations (red) region for Design III. The fraction of sustained oscillations for the three models as it results from this figure is: 0.0709 (Design I), 0.0789 (Design II)and 0.1664 (Design III), which puts Design III as the most "robust" to changes in the parameters of the three. However, rather than associating such a quantitative robustness measure, a better perspective on the distinctive characteristics of the oscillations for the three models is required. The features are represented in Figure S8.5 showing the distribution of amplitude and periods. The periods have been represented in hours, and it can be seen that Δ sets the temporal scale of the oscillations. Lower values of Δ are expected to move the highest peak i the distributions to lower values, if oscillatory cases exist for lower Δ. The figure also shows how the abundance of SNIC-bifurcation cases for Design I translates into a queue of long periods. This is contrasted by the Design III which presents a very characteristic period. While the distribution of the repressor amplitude is similar for the three models, the activator's amplitude is differently implemented by the three models (upper panels).

---

[1] Guantes R, Poyatos JF: **Dynamical principles of two-component genetic oscillators.** *PLoS Comput Biol* 2006, **2**(3):e30, [http://dx.doi.org/10.1371/journal.pcbi.0020030].

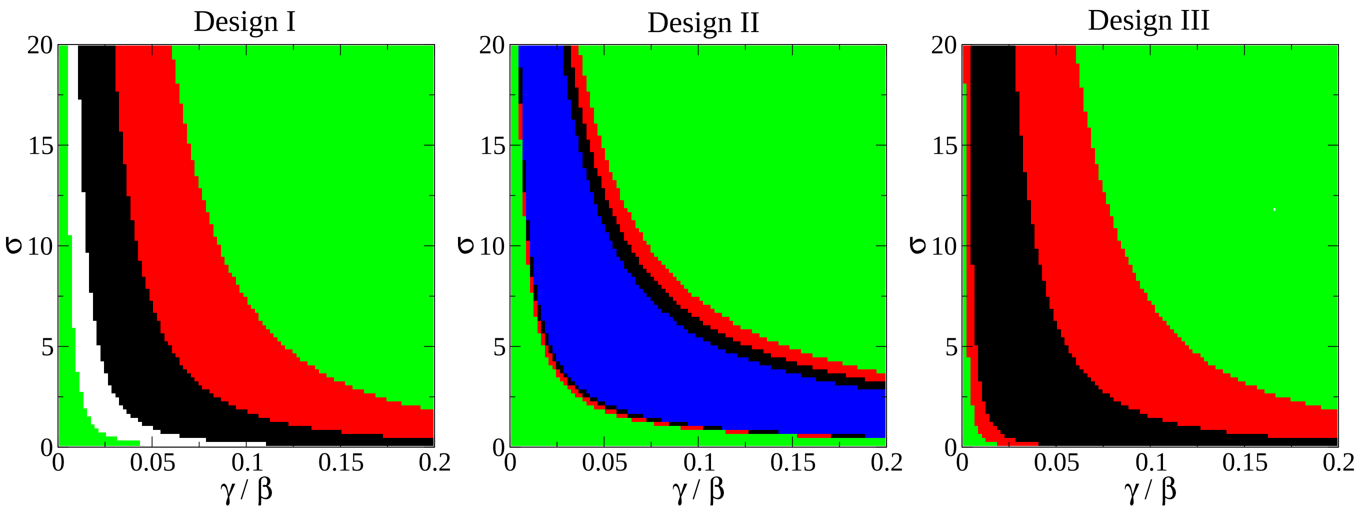

Figure S8.1: Dynamical behavior of the three designs for $\Delta = 10$ as a function of repressor strength, σ and ratio of protein production, γ/β. Green regions correspond to cases of unique stable fixed point; white, to cases of three fixed points; black, to unique fixed unstable point with eigenvalue of non-zero imaginary part; blue, to unique fixed unstable point with eigenvalue of zero imaginary part; red, to unique fixed stable point with eigenvalue of non-zero imaginary part. *Sustained* oscillations exist for black and blue regions, while *damped* oscillations, for the red regions.

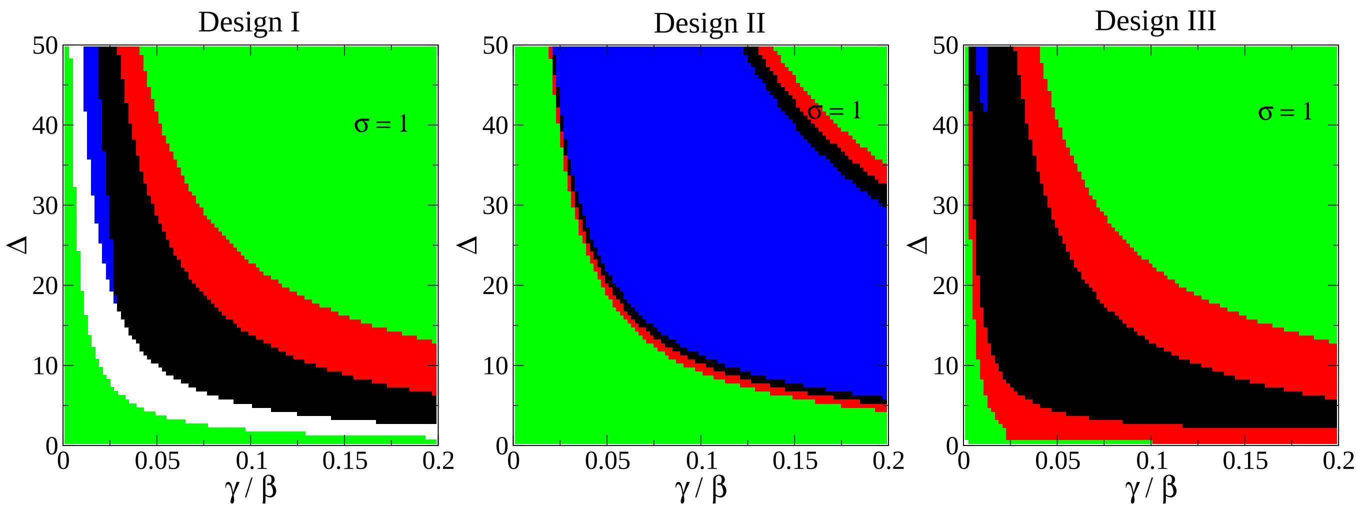

Figure S8.2: Similar to Figure S8.1, but for the parameter space $(\gamma/\beta, \Delta)$ for fixed $\sigma = 1$.

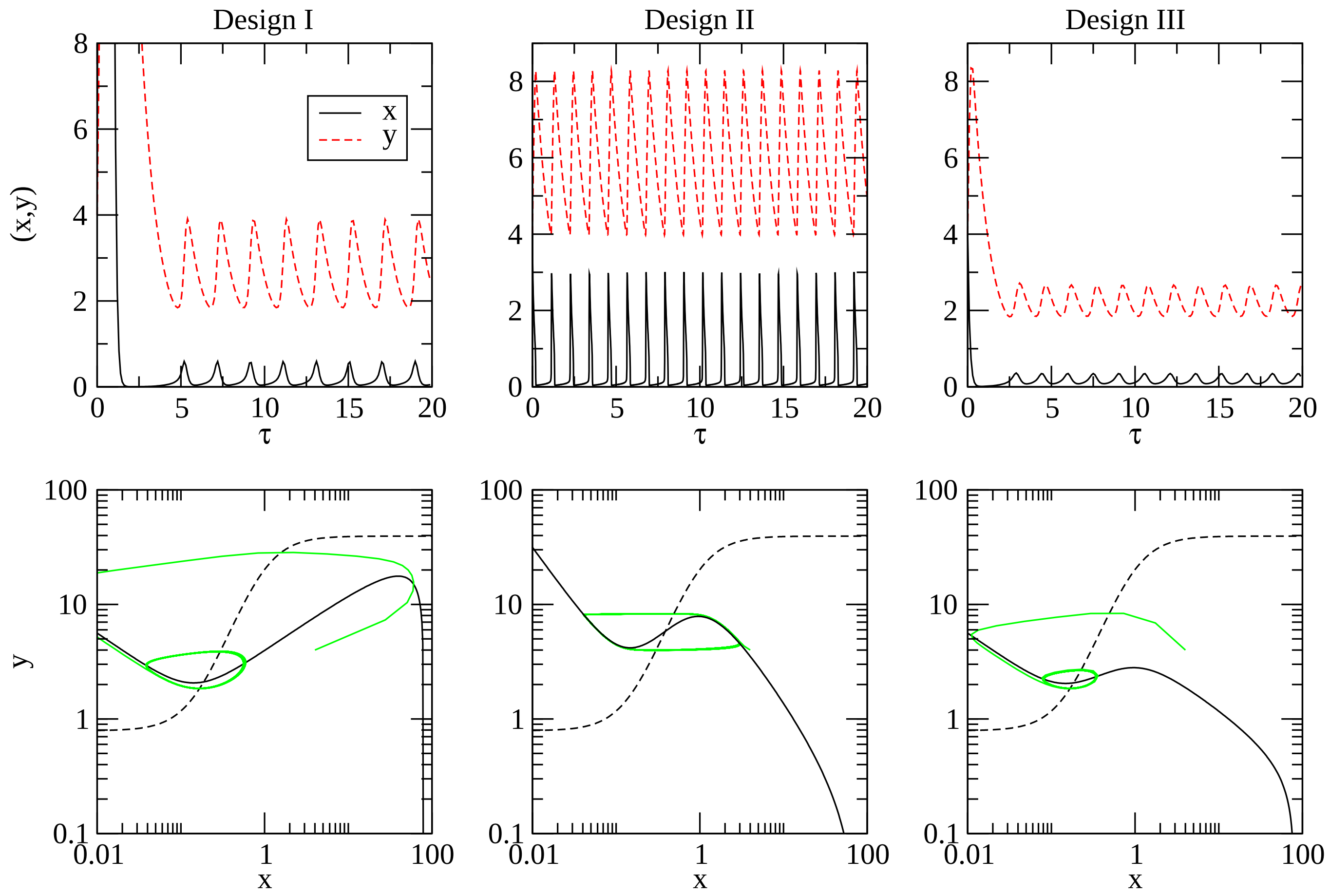

Figure S8.3: An example of timeseries of oscillations for the three designs for $\alpha = 50$, $\beta = 1.58$, $\gamma = 0.079$, $\Delta = 10$, $\sigma = 5$ (see location in Figure S8.1). Notice the spiky features of the Design II oscillations. Notice also the difference in the period and amplitude for the three designs, a feature that needs to be considered at the moment of choosing which design would be easier to implement experimentally. The lower panels illustrate the nullclines for the three cases (solid black line, y_x; dashed line, y_y), together with the trajectory (in green) from the upper panels.

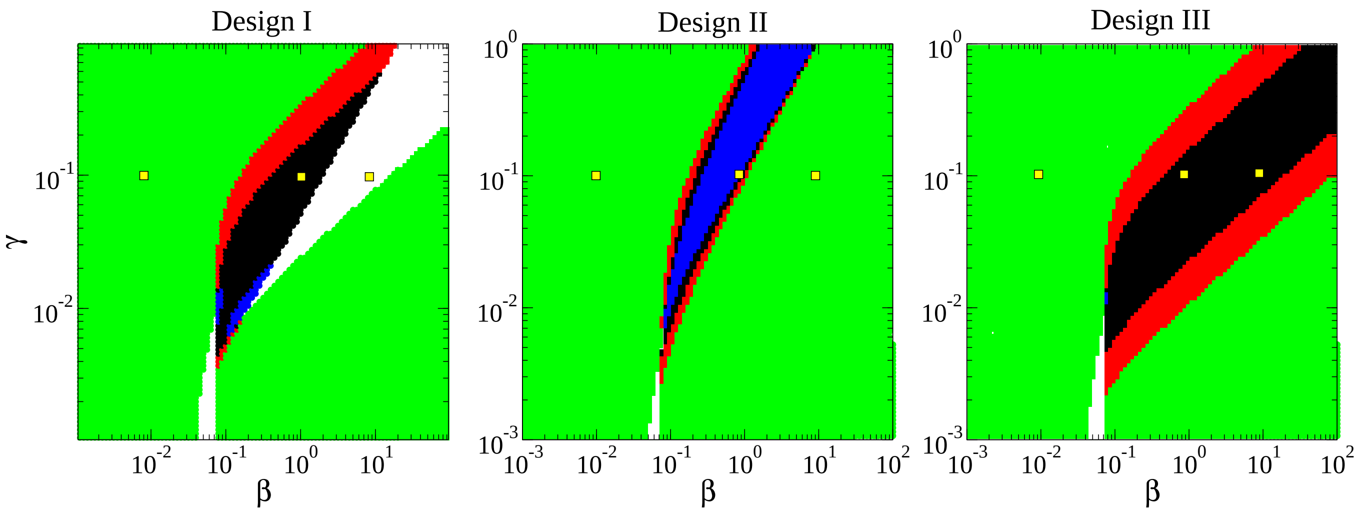

Figure S8.4: Similar to Figures S8.1 and S8.2, but for the space (β, γ) with $\alpha = 50$, $\Delta = 10$, $\sigma = 1$, as in Figure 3 from the main text to which we refer. Notice the extension of the black-white border for Design I associated to the SNIC bifurcation. This fact influences the period distribution and the shape of the oscillations. The yellow boxes represent three examples chosen for the discussion of the nullclines in Additional File 7 (Figure S7.3).

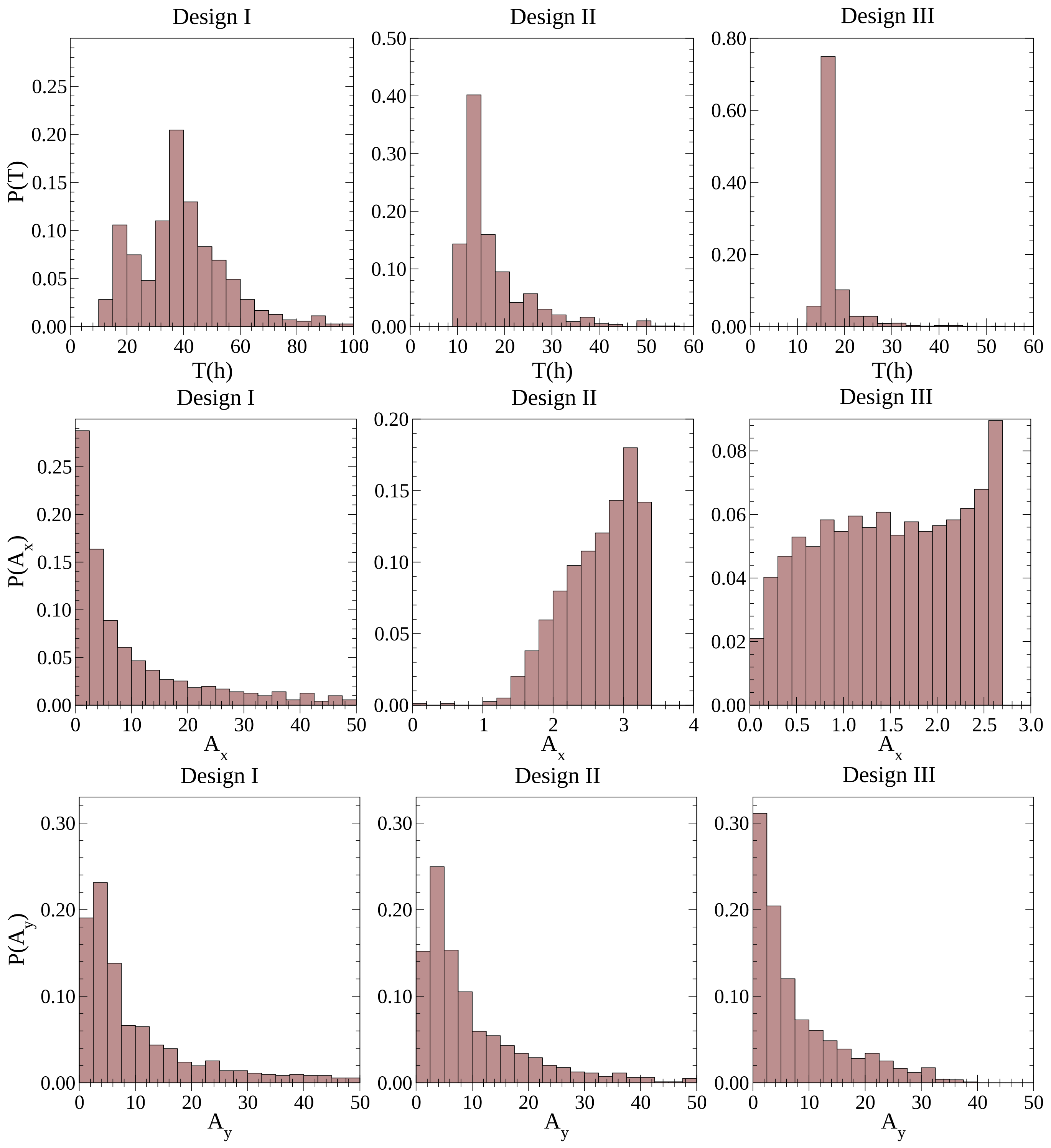

Figure S8.5: The features of the oscillatory solutions from Figure S8.4. It shows the distribution of periods in hours (upper panels), the non-dimensional amplitude for the activator (middle panels) and for the repressor (lower panels). The period is determined as the interval between maxima.
