# Supplementary material for: Avoiding transcription factor competition at promoter level increases the chances of obtaining oscillation
Source: BMC Syst Biol. 2010 May 17;4:66. doi: 10.1186/1752-0509-4-66 (PMC2898670; doi:10.1186/1752-0509-4-66)
Supplement: Additional file 9 — Dynamical differences between the designs: discussion of the nullclines. [file 1752-0509-4-66-S9.PDF]

Additional File 9 of
Avoiding transcription factor competition at promoter level increases the chances of obtaining oscillations

Nullcline analysis

The nullclines corresponding to the three designs are

Nullclines : Design I

$$y_x = \sqrt{\frac{1}{\sigma}\left(\beta\frac{1+\alpha x^2}{x} - x^2 - 1\right)}$$
$$y_y = \Delta\gamma\frac{1+\alpha x^2}{1+x^2}$$

Nullclines : Design II

$$y_x = \frac{1}{\sigma}\left(\beta\frac{1+\alpha x^2}{x(1+x^2)} - 1\right)$$
$$y_y = \Delta\gamma\frac{1+\alpha x^2}{1+x^2}$$

Nullclines : Design III

$$y_x = \sqrt{\frac{1}{\sigma}\left(\beta\frac{1+\alpha x^2}{x(1+x^2)} - 1\right)}$$
$$y_y = \Delta\gamma\frac{1+\alpha x^2}{1+x^2}$$

Figure S9.1 shows an example of typical case of oscillations for Design I and III, and the corresponding nullclines. Panel B also includes the nullclines for Design II, for comparison. Figure S9.2 shows an example in which Design I has lost the oscillation capability as the system presents now three fixed points: two stable ones separated by an unstable one. In panel B it can be seen that, while Design III still shows oscillations, even though of low amplitude, Design II has lost this capability, as the fixed point (on green line) is situated outside the region of rapid change of the sigmoidal y_y.

Besides these examples, we better illustrate the capability of displaying oscillations in the three designs in Figure S9.3. It includes three parameter sets that constitute the crossing of the instability region in Figure 3 in the main text or Figure S8.4 at fixed value of γ. In order to present oscillations, all three systems must have a unique fixed point and it must be situated in the region of fast increase of y_y. In this region of rapid increase, small changes in concentration are amplified and can give rise to oscillations. From Figure S9.3 can be see that, while Design I looses the oscillations by acquiring new fixed points (SNIC bifurcation), the other two designs loose it by the exit of the fixed point from the region of rapid change in y_y. Let us pay closer attention to Designs II and III. From the similarity between their y_x ($f(x)$ for Design II and $\sqrt{f(x)}$ for Design III), one can expect that they might share dynamical features. In the following, we have explored these features. Notice that for β greater than a certain threshold, y_x for both designs shows a minimum and a maximum (small arrows), and their existence makes oscillations possible. The x-alue of these points is the major factor responsible for the activator's amplitude in the associated oscillations: the further apart they are, the greater the activator's amplitude. In this sense, it is interesting to notice that the x value of these points depends solely on α and is the same for both designs: $\sqrt{(\alpha - 3 \pm \sqrt{\alpha^2 - 10\alpha + 9})/2\alpha}$. This information also tells us that by increasing β, the nullclines y_x are simply displaced upwards, without changing the shape. For this reason, these two designs present a characteristic frequency and a limitation in x amplitude.

Moreover, from the equations can thus be seen that, starting from an oscillation-producing parameter case, an increase in β implies upwards displacement of y_x and an increase in Δ or γ implies upwards displacement of y_y. For this reason, by increasing both β and γ simultaneously, their crossing is maintained and thus the oscillations too. This does not occur for Design I for which a change in β implies not only a displacement, but a change in shape, leading to a three-crossing case: no oscillations. Design I differs in this aspect from the other two designs, as the y_y nullclines presents extrema that depend on the values of β, not only on α, and thus their position changes with β. As β increases, the two extrema become more separated and the maximum crosses y_y, at which point oscillations disappear through the SNIC bifurcation. Moreover, the shape of the y_x function presents a high dynamic range between these extrema, fact that leads to high amplitudes in both x and y, as can be see in Figure S8.5. If oscillations are lost through the SNIC bifurcation, to recover them, y_y can be displaced upwards by increasing γ. This explains the oblique black-white frontier in Figure S8.4 for Design I. By increasing both γ and β, a point is reached at which γ value is not enough for ensuring a unique crossing instead of three, and oscillations are not possible even by increasing γ even further. At this point, the difference in y between the two extrema of y_x is larger than the y-range of y_y.

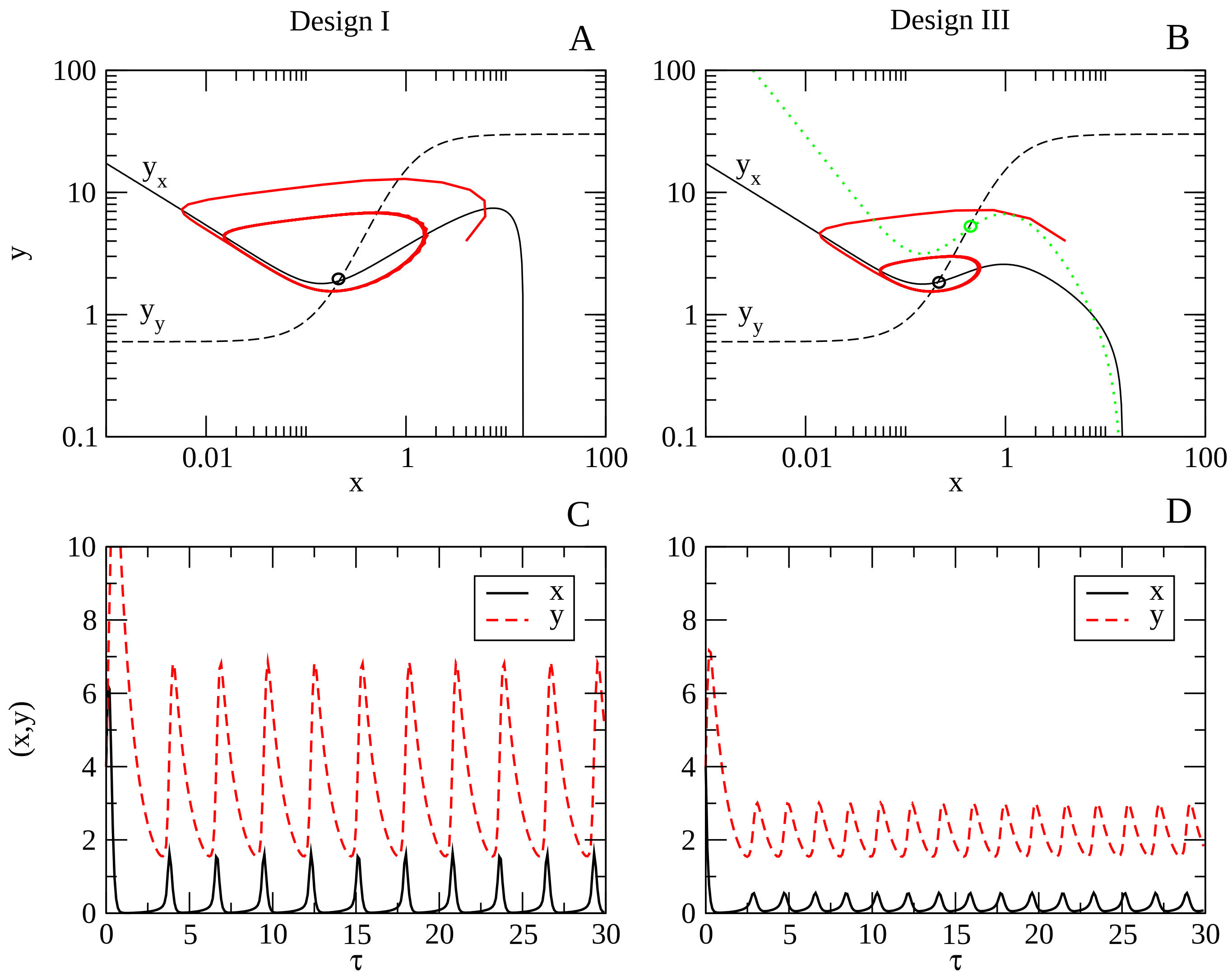

Figure S9.1: The nullclines for the case $\beta = 0.3, \gamma = 0.06, \Delta = 10$ for Design I (left) and III (right). The notations and style of the figure are similar to those from Figure 2 of Guantes&Poyatos(2006) for an easier comparison: (A) and (B) show the nullclines y_x of the activator (solid line) and y_y of the repressor (dashed line), as well as the limit cycle trajectory in red. The intersection points of the nullclines represent the fixed points of the system and are represented in the figure by circles. The dotted green line is the nullcline y_x corresponding to Design II with the same parameter values. (C) and (D) show the time series for the activator, x and repressor, y.

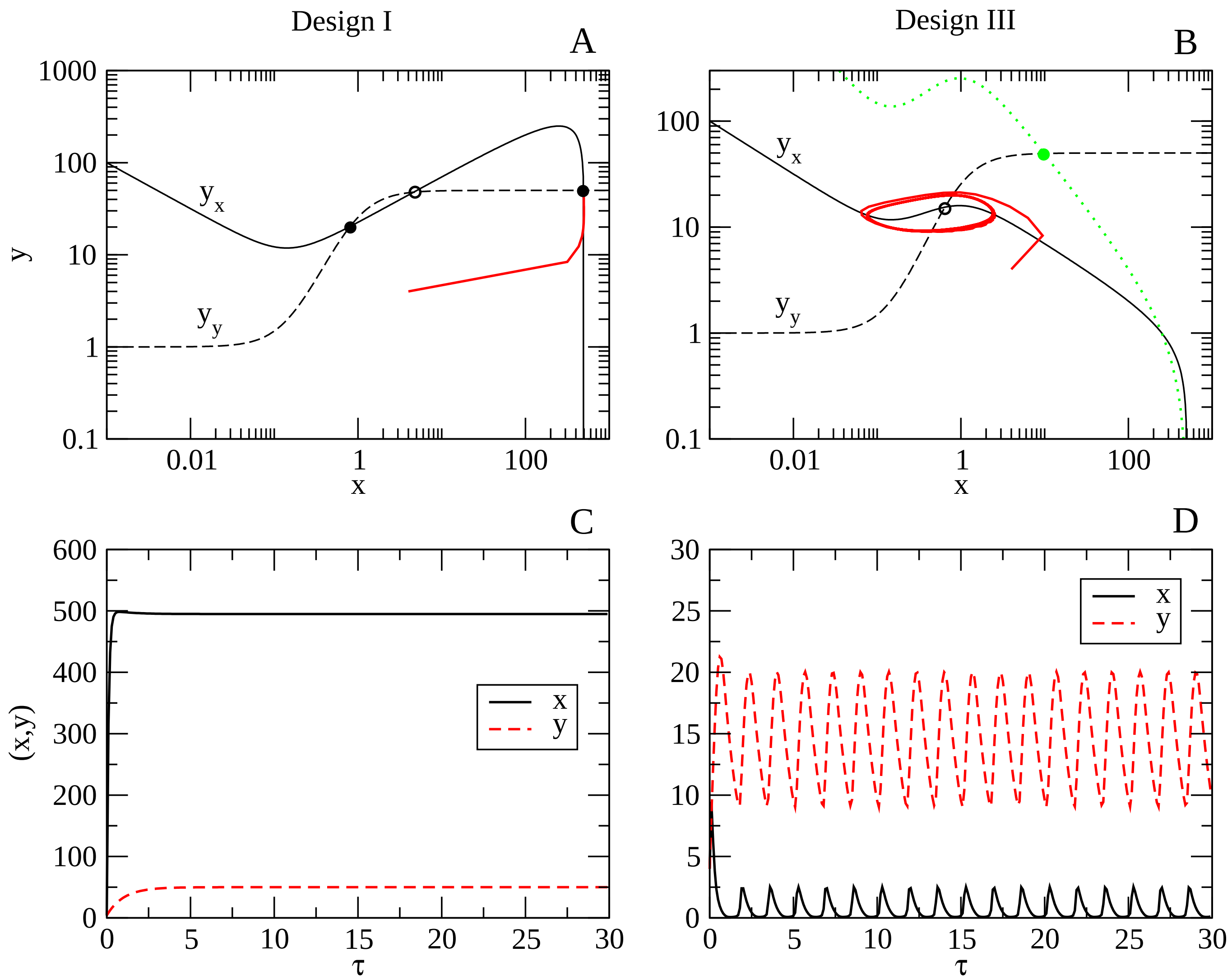

Figure S9.2: The nullclines for the case $\beta = 10, \gamma = 0.1, \Delta = 10$ for Design I (left) and III (right), with notations as in Figure S9.1. Notice how the nullclines for Design I presents three intersection points representing the three fixed points of the system. The filled circles denote stable fixed points, while empty circles denote unstable fixed points. The dotted line is the nullcline y_x corresponding to Design II with the same parameter values. For Design II, this case does not lead to oscillations.

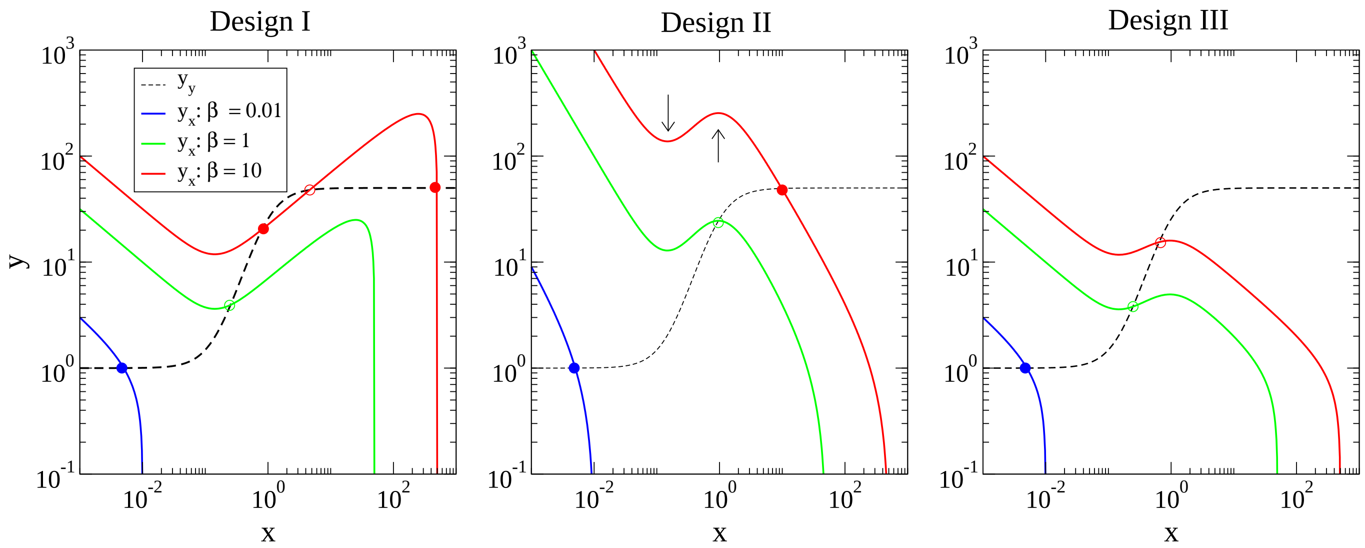

Figure S9.3: Visualization of the nullclines for the three designs corresponding to three representative cases (yellow boxes in Figure S8.4, to which we refer for the justification of the parameters choice): $\alpha = 50,\ \gamma = 0.01,\ \Delta = 10,\ \sigma = 1$, and three value of β: 0.01, 1, 10.
