# Supplementary material for: Avoiding transcription factor competition at promoter level increases the chances of obtaining oscillation
Source: BMC Syst Biol. 2010 May 17;4:66. doi: 10.1186/1752-0509-4-66 (PMC2898670; doi:10.1186/1752-0509-4-66)
Supplement: Additional file 10 — The influence of internal noise. [file 1752-0509-4-66-S10.PDF]

Additional File 10 of
Avoiding transcription factor competition at a promoter level increases the chances of obtaining oscillations

Influence of noise

We present here the results on stocahstic simulations which take into account the internal noise of the chemical system. In chemical systems, internal noise results from a low number of molecules at cellular level. One of the widely employed simulation algorithms was introduced by Gillespie [1], and stochastically determines what is the next chemical reaction to occur in the predefined reactions system and when it occurs, according to the transition probability associated to each reaction event. However, this method is time-consuming in the case of large systems, and also requires the knowledge of all the chemical reactions in detail. Another method to study internal noise, also proposed by Gillespie, is the Chemical Langevin (CL) method [2], which was proved to be a good approximation of the exact algorithm mentioned above. Here we have employed the latter method.

Below we present the application of the method for Design I, while the associated equations for Design II and Design III can be obtained in a similar manner. The equations describing the deterministic evolution of the Design I as defined in the main manuscript are:

$$\frac{dx}{d\tau} = \Delta\beta\frac{1}{1+x^2+\sigma y^2} + \Delta\beta\frac{\alpha x^2}{1+x^2+\sigma y^2} - \Delta x \equiv a_1 + a_2 - a_3 \quad (1)$$

$$\frac{dy}{d\tau} = \Delta\gamma\frac{1}{1+x^2} + \Delta\gamma\frac{\alpha x^2}{1+x^2} - y \equiv a_4 + a_5 - a_6 \quad (2)$$

which, by means of the CL method become the stochastic equations

$$\frac{dx}{d\tau} = a_1 + a_2 - a_3 + \frac{1}{\sqrt{\Omega}}(\sqrt{a_1}\xi_1(t) + \sqrt{a_2}\xi_2(t) - \sqrt{a_3}\xi_3(t)) \quad (3)$$

$$\frac{dy}{d\tau} = a_4 + a_5 - a_6 + \frac{1}{\sqrt{\Omega}}(\sqrt{a_4}\xi_4(t) + \sqrt{a_5}\xi_5(t) - \sqrt{a_6}\xi_6(t)) \quad (4)$$

where Ω is the size of the system ($N_A V$, with N_A Avogadro's number, and V, the volume in l), while $\xi_1 - \xi_6$ are uncorrelated Gaussian white noise with zero mean. One can see that, as the volume increases, the terms in parentheses become neglectable, and the deterministic case is recovered.

Similar CL equations can be constructed for Design II adn Design III. An example of stochastic simulations appears in Figure S9 for cases close to a bifurcation point. In this representation we have also followed [3] (Supporting Information File) in order to refer to their results. It can be seen from this figure that the period distribution for Design I and II exhibits a long tail, characteristic to noise-induced oscillations, as discussed by [3]. On the contrary, Design III shows a narrow period distribution representative of the Hopf bifurcation where oscillations of a given frequency emerge and maintain the frequency after the bifurcation point. Interestingly, Design II also presents a Hopf bifurcation, but its range is very narrow (black region in Figure S8.2 middle panel), with the eigenvalue rapidly losing the complex part (responsible for the frequency) and the oscillations become typical of relaxation oscillator (spiky). We notice a difference between our results and those of [3] as presented in their Supporting Information with respect to Design II. In the case of the Hopf bifurcation of Design III, it has already been observed for other systems [4] that internal noise lowers the bifurcation point and thus allows oscillations for parameters values for which the determinsitic case does not. It is thus a question of determining the appropriate level of modeling for the system under study: is the noise expected to play a substantial role in the system? Is it expected to be functioning at low number of molecules? In synthetic biology the level of noise can be lowered by employing strong promoters with high transcription rate.

---

[1] Gillespie DT: **Exact Stochastic Simulation of Coupled Chemical Reactions**. *J. Phys. Chem.* 1977, **81**(25):2340–2361.
[2] Gillespie DT: **The chemical Langevin equation**. *J. Chem .Phys.* 2000, **113**:297–306.

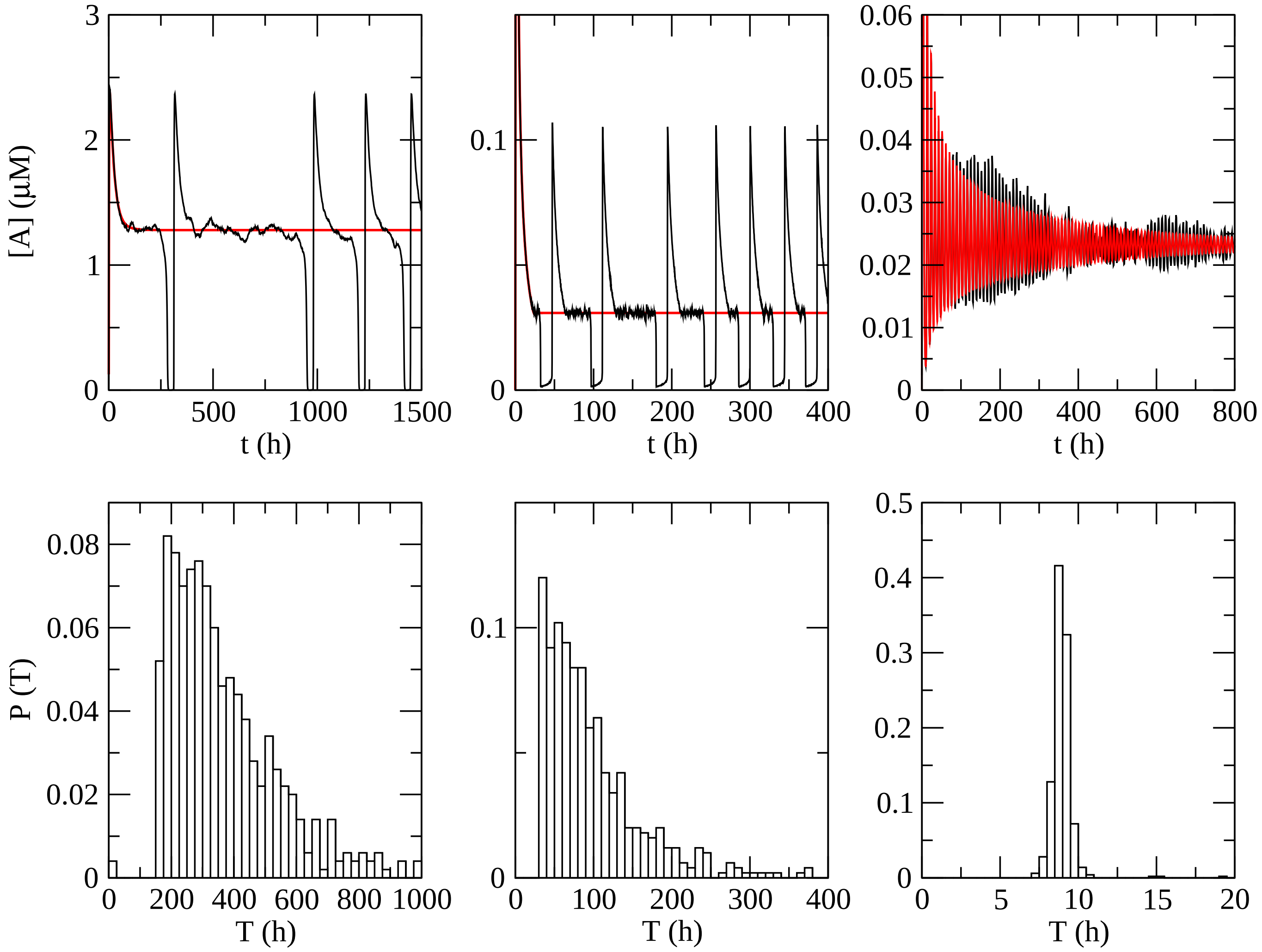

Figure S10: Analysis of the distribution of oscillation periods for stochastic cases of: $\alpha = 50$, $\beta = 1.58$, $\gamma = 0.079$, $\sigma = 1$, and : $\Delta = 10$, $\Omega = 5$ for Design I, $\Delta = 20$, $\Omega = 50$ for Design II and $\Delta = 4.3$, $\Omega = 10$ for Design III. Upper panels show an example of timeseries with stochastic simulations (black) and the deterministic ones (red). Lower pannels show the distribution of periods for the three designs, where a period was considered the interval between maxima. See also Supporting information of [3].

[3] Guantes R, Poyatos JF: **Dynamical principles of two-component genetic oscillators.** *PLoS Comput Biol* 2006, **2**(3):e30, [http://dx.doi.org/10.1371/journal.pcbi.0020030].

[4] Hou Z, Xin H: **Internal noise stochastic resonance in a circadian clock system**. *J. Chem. Phys.* 2003, **119**:11508–11512.
